# Comprehensive evaluation of targeted multiplex bisulphite PCR sequencing for validation of DNA methylation biomarker panels

**DOI:** 10.1186/s13148-020-00880-y

**Published:** 2020-06-22

**Authors:** Dilys Lam, Phuc-Loi Luu, Jenny Z. Song, Wenjia Qu, Gail P. Risbridger, Mitchell G. Lawrence, Jennifer Lu, Matt Trau, Darren Korbie, Susan J. Clark, Ruth Pidsley, Clare Stirzaker

**Affiliations:** 1 Epigenetics Research Laboratory, Genomics and Epigenetics Division, Garvan Institute of Medical Research, Sydney, New South Wales 2010 Australia; 2 St Vincent’s Clinical School, UNSW Sydney, Sydney, New South Wales 2010 Australia; 3 Monash Partners Comprehensive Cancer Consortium, Monash Biomedicine Discovery Institute Cancer Program, Prostate Cancer Research Group, Department of Anatomy and Developmental Biology, Monash University, Clayton, VIC 3800 Australia; 4 Cancer Research Division, Peter MacCallum Cancer Centre, Melbourne, VIC 3000 Australia; 5 Sir Peter MacCallum Department of Oncology, The University of Melbourne, Parkville, VIC 3010 Australia; 6 Centre for Personalised Nanomedicine, Australian Institute for Bioengineering and Nanotechnology (AIBN), The University of Queensland, St Lucia, Queensland 4072 Australia

**Keywords:** DNA methylation, Methylome, Epigenetics, Multiplex, Bisulphite amplicon sequencing, Clinical biomarkers

## Abstract

**Background:**

DNA methylation is a well-studied epigenetic mark that is frequently altered in diseases such as cancer, where specific changes are known to reflect the type and severity of the disease. Therefore, there is a growing interest in assessing the clinical utility of DNA methylation as a biomarker for diagnosing disease and guiding treatment. The development of an accurate loci-specific methylation assay, suitable for use on low-input clinical material, is crucial for advancing DNA methylation biomarkers into a clinical setting. A targeted multiplex bisulphite PCR sequencing approach meets these needs by allowing multiple DNA methylated regions to be interrogated simultaneously in one experiment on limited clinical material.

**Results:**

Here, we provide an updated protocol and recommendations for multiplex bisulphite PCR sequencing (MBPS) assays for target DNA methylation analysis. We describe additional steps to improve performance and reliability: (1) pre-sequencing PCR optimisation which includes assessing the optimal PCR cycling temperature and primer concentration and (2) post-sequencing PCR optimisation to achieve uniform coverage of each amplicon. We use a gradient of methylated controls to demonstrate how PCR bias can be assessed and corrected. Methylated controls also allow assessment of the sensitivity of methylation detection for each amplicon. Here, we show that the MBPS assay can amplify as little as 0.625 ng starting DNA and can detect methylation differences of 1% with a sequencing coverage of 1000 reads. Furthermore, the multiplex bisulphite PCR assay can comprehensively interrogate multiple regions on 1–5 ng of formalin-fixed paraffin-embedded DNA or circulating cell-free DNA.

**Conclusions:**

The MBPS assay is a valuable approach for assessing methylated DNA regions in clinical samples with limited material. The optimisation and additional quality control steps described here improve the performance and reliability of this method, advancing it towards potential clinical applications in biomarker studies.

## Introduction

DNA cytosine methylation is a key epigenetic mark associated with gene regulation and function [1, 2]. DNA methylation can be modified by environmental exposures [3, 4] and is associated with a wide range of diseases including developmental pathologies [5] and cancer [6–8], where methylation changes are particularly pronounced. The growing body of public DNA methylation datasets for a variety of cancer types [9–11] provides data to enable discovery of novel clinical biomarkers for both early detection of tumours and monitoring of minimal residual disease [12–14]. The use of DNA methylation as a clinical biomarker is made feasible by the fact that it is highly stable and retained during long-term storage of clinical material, including formalin-fixed paraffin-embedded tissue (FFPET).

The first stage of DNA methylation biomarker discovery is usually to screen the genome for methylation changes associated with the clinical phenotype of interest. These studies employ epigenome-wide methods that generate data at single-base resolution, such as whole-genome bisulphite sequencing (WGBS) [15–18] and microarray technologies [19, 20]. Following analysis, DNA methylation differences are frequently observed between disease groups, often occurring across adjacent CpG sites, termed differentially methylated regions (DMRs) [21]. Translation of these findings into the clinic requires further screening and validation of the DMRs in independent retrospective and prospective cohorts to assess their clinical value as a biomarker for the phenotype of interest. This necessitates the development of DNA methylation assays that are compatible with, and easily integrated into, routine clinical use, thus needing to be cost-effective, scalable and reproducible [22, 23]. Additionally, clinical samples are often limited; therefore, the method needs to produce accurate methylation data from low and degraded DNA sample inputs. To this end, it has been previously shown that loci-specific PCR-based methods, such as targeted bisulphite PCR sequencing, exhibit the most consistent performance on low-input clinical samples compared to other DNA methylation assays, such as padlock probe-based or microdroplet-based enrichment techniques [22]. Furthermore, PCR primers can be multiplexed to produce multiple amplicons in a single bisulphite PCR reaction. This allows the interrogation and generation of methylation data across many regions concurrently in one experiment. Together, this establishes multiplex bisulphite PCR sequencing (MBPS) as a technology ready for widespread biomarker development and clinical use.

An MBPS assay that is able to deliver robust methylation data from FFPET clinical DNA has previously been developed and published [23]. Here, we provide an improved protocol and additional optimisation steps for this methodology. We perform technical comparisons between multiplex bisulphite PCR sequencing and the WGBS platform. We comprehensively evaluate its utility in interrogating multiple genomic regions simultaneously, in minimal amounts of FFPET clinical DNA and in patient-derived circulating cell-free DNA (cfDNA). Finally, we demonstrate the ability of the MBPS assay to measure intra- and inter-sample methylation variability through assessment of epigenetic heterogeneity.

## Results

Previously, we performed DNA methylation biomarker discovery studies using whole-genome methylation profiling of prostate cancer [24] and breast cancer [25]. The DMRs identified in these studies form the basis of the biomarker panels of multiplex PCR primers used in the current work. We designed two panels of multiplex primers each for prostate cancer—63 DMRs (panel 1: *n* = 31 and panel 2: *n* = 32 PCR amplicons in each panel)—and for breast cancer—33 DMRs (panel 1: *n* = 17 and panel 2: *n* = 16 PCR amplicons in each panel) (see the “Material and methods”). We use these panels to demonstrate the steps required for panel optimisation for MBPS and to evaluate the performance of the assay, as described below.

### Overview of multiplex bisulphite PCR sequencing protocol

**Fig. 1:**
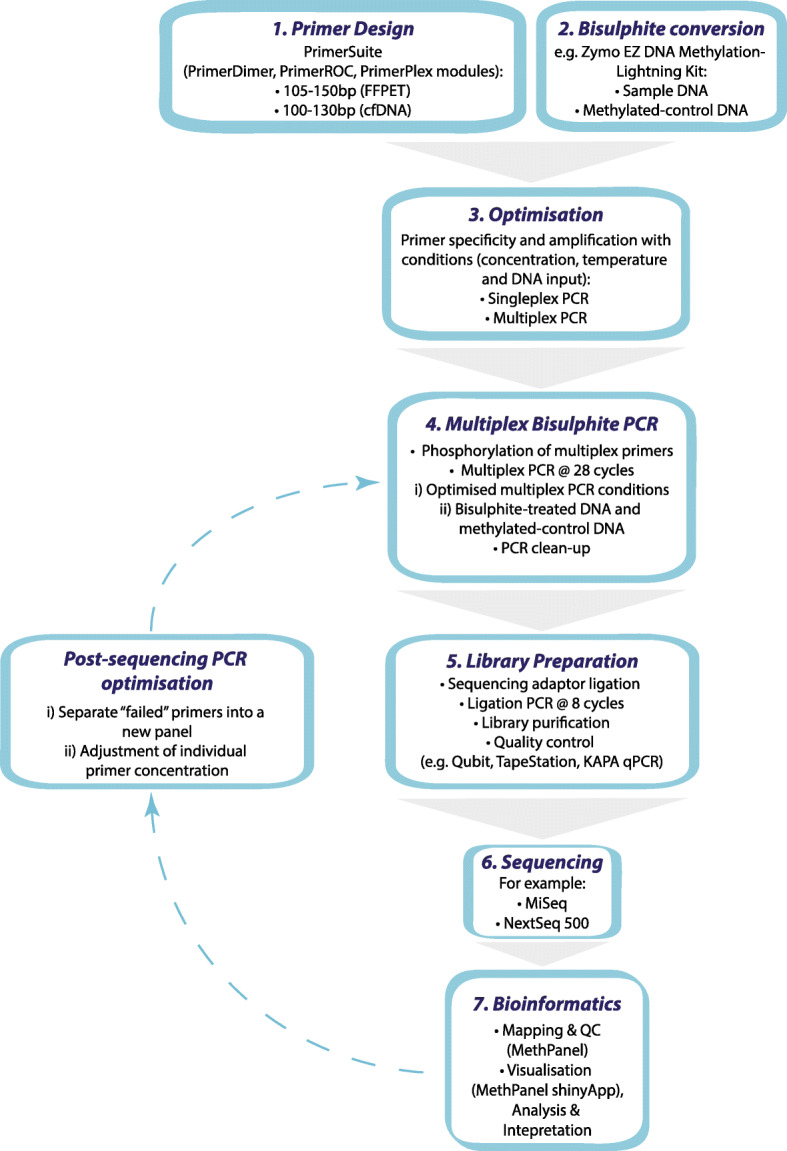
Flow diagram of the multiplex PCR bisulphite sequencing assay. A flow diagram highlighting the key steps in the multiplex PCR bisulphite sequencing assay. A detailed step-by-step protocol is included in Supplementary Information (Additional file 2)

An overview of the MBPS protocol is shown in Fig. 1, comprising the following key steps: (1) primer design: design primers for the user’s genomic regions of interest. For this, we recommend the multiplex-friendly primer design software PrimerSuite [26]. (2) Bisulphite conversion: perform bisulphite conversion of DNA. This converts unmethylated cytosines into uracils, thus allowing methylated and non-methylated CpGs to be distinguished following PCR and sequencing. Whilst optimising the assay, it is advised to use ‘test’ DNA rather than DNA from precious clinical samples. (3) Optimisation: perform PCR optimisation to ensure that all of the primers are amplifying bisulphite-converted DNA as expected. Parameters include annealing temperature, primer concentration and DNA input amount. Optimisation is performed first with individual primer pairs (‘singleplex PCR’) and then with multiplex panels of pooled primer pairs. (4) Multiplex bisulphite PCR: perform multiplex bisulphite PCR on bisulphite-treated DNA of the samples deemed necessary to assess the performance of the method after sequencing. (5–7) Library preparation, sequencing and bioinformatics: perform library preparation, purification and quantification, followed by sequencing and bioinformatic processing and analysis. This can be performed using our dedicated bioinformatic mapping and QC pipeline, called *MethPanel* [27], which includes data visualization using the shinyApp (https://github.com/thinhong/MethPanel). The sequencing results may reveal that further optimisation is needed, in which case post-sequencing multiplex bisulphite PCR optimisation (as described below) can be conducted and sequencing repeated (steps 4–7) to confirm the good quality of sequencing data, before applying the method to clinical samples and appropriate controls. A detailed version of the flow-diagram (Additional file 1: Figure S1) and a step-by-step protocol (Additional file 2) is provided in the supplementary materials.

### Optimisation of panels for multiplex bisulphite PCR sequencing

#### Pre-sequencing PCR optimisation

**Fig. 2:**
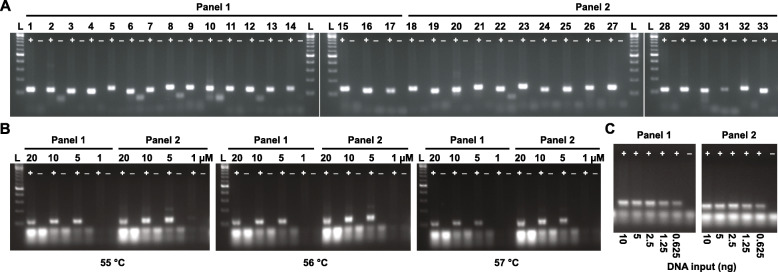
Pre-sequencing optimisation of multiplex PCR primers. **a** PCR products of singleplex amplification of 33 individual primer pairs from the breast cancer panels run on 2% agarose gel. The gels show the specificity of all the primer pairs and PCR products of the correct size (100–130 bp) with minimal primer dimer formation. (−) no template; (+) bisulphite-treated test DNA template (10 ng); (L) 100 bp DNA Ladder. **b** Singleplex primers were pooled into their respective multiplex panels, and the outcome of multiplex PCR reactions is shown at different primer concentrations (20 μM, 10 μM, 5 μM and 1 μM) and at different annealing temperatures (55 °C, 56 °C and 57 °C). **c** PCR products from the multiplex panels testing DNA input amounts of 10 ng, 5 ng, 2.5 ng, 1.25 ng and 0.625 ng of bisulphite-treated control DNA; (+) test DNA; (−) no template control

PCR conditions need to be optimised to ensure good amplification of target regions using each individual primer pair in a panel. Bisulphite-treated DNA is PCR-amplified for each primer pair as a singleplex PCR to verify primer specificity and minimal primer dimer formation. For example, during singleplex PCR optimisation of the breast cancer panels on ‘test’ DNA, we saw robust, specific amplification from all individual primer pairs, with only one primer pair (#31) showing a slightly reduced yield of PCR product (Fig. 2a). Next, the primers are pooled into their respective multiplex panels, and the optimal primer concentration and PCR cycling temperature are determined. Here, we show that for the breast panels, a concentration of 20 μM for each primer pool yields excessive primer dimer relative to lower primer concentrations (1, 5 and 10 μM) whilst a concentration of 1 μM did not amplify the DNA (Fig. 2b). Temperatures of 55 °C and 56 °C both yield robust PCR amplification. Thus, we used a temperature of 56 °C and a primer concentration of 10 μM in all subsequent PCRs with the breast cancer panels. As these assays were to be performed on DNA from limited breast cancer samples, a titration of DNA input concentrations (10 ng to 0.625 ng per multiplex PCR) was run to assess the minimum amount of DNA required for amplifying enough DNA for library preparation (Fig. 2c). Both breast cancer panels successfully amplified as little as 0.625 ng input DNA. Similar images for the prostate cancer panels are supplied in the supplementary materials (Additional file 3: Figure S2).

#### Post-sequencing quality control and optimisation

Following library preparation, DNA sequencing and analysis, further refinement and optimisation of the MBPS assay may need to be performed post-sequencing, as described below:

##### Sequencing coverage

**Fig. 3:**
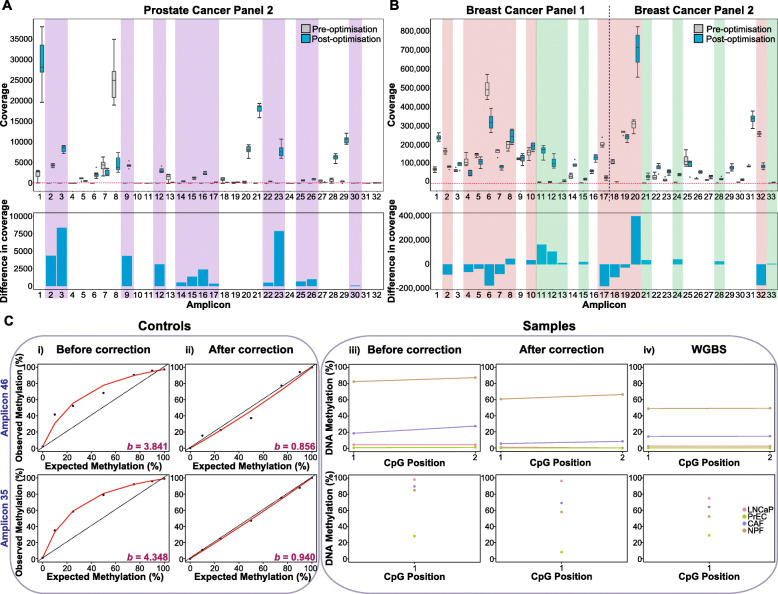
Post-sequencing optimisation of multiplex bisulphite PCR assay. Boxplots show the range of sequencing coverage for individual amplicons in the **a** prostate cancer and **b** breast cancer panels. Light grey and blue boxes are used to depict the sequencing coverage before and after post-sequencing optimisation respectively. The purple background in **a** highlights the amplicons that originally failed sequencing (i.e. coverage < 100). The primers for these amplicons were pooled and re-amplified in an individual third multiplex panel. **b** For the breast cancer panels—the green background indicates the amplicons that originally failed sequencing and the red background indicates the amplicons that were originally amplified more than needed. For these primer pairs, primer concentrations were doubled (20 to 40 μM) or halved (20 to 10 μM) respectively to achieve more uniform amplicon coverage. The corresponding barplot shows the change in sequencing coverage before and after post-sequencing optimisation. **c** (i) PCR bias is introduced by PCR amplification of 2 example prostate amplicons (amplicon 46 and 35) using methylated-control DNAs (0%, 10%, 25%, 50%, 75%, 90%, 100%). Observed methylation after amplification (*y*-axis) is plotted against expected methylation levels (*x*-axis). Regression analysis was used to calculate a value of bias (*b*) as described by Warnecke et al. [28]. Red line = line of best fit from the regression; dotted line = line of best fit if data was unbiased (i.e. *b* = 1). (ii) The result of PCR-bias correction by regression on the methylated control DNA. The corrected methylation level (*y*-axis) is plotted against the expected methylation level (*x*-axis) showing that PCR bias has been effectively corrected. (iii) Multiplex bisulphite PCR methylation values for four biological samples are corrected for PCR bias based on the calculated bias value from (i) (light pink = LNCaP, light green = PrEC, violet = CAF, light brown = NPF). The corrected values are more similar than the uncorrected values to the same samples profiled by WGBS (iv)

An initial quality control step is to compare the sequencing coverage of different amplicons to confirm uniform amplification. This is important because with such a large number of primers competing to hybridise and amplify DNA in one PCR, there are likely to be primers that fail to amplify completely (i.e. amplicons that have very low or no coverage) or primers that favourably amplify over others. This may occur despite using the multiplex-specific primer design software and pre-sequencing PCR optimisation. These deviating primers can either be (1) removed from future multiplex PCR reactions, (2) redesigned or (3) potentially ‘rescued’. For example, we observed that the second prostate cancer multiplex panel had a number (*n* = 13/32) of dropouts (i.e. coverage < 100) (Fig. 3a). We took these 13 ‘failed’ primers (Fig. 3a, grey boxes with purple background) and pooled them together as a separate, ‘new’ multiplex panel. We then performed the optimisation of the multiplex PCR (primer concentration and temperature), observing that with a 3-fold increase in primer concentration (as compared to the original two prostate panels), we were able to amplify these regions in a separate multiplex PCR reaction. Sequencing of this new sub-panel showed that all the amplicons now had sufficient sequencing coverage for analysis, with the exception of amplicon #30 (Fig. 3a, blue boxes with purple background). An alternative way to improve the coverage of individual amplicons is to leave primers grouped with the primers from their original panels and adjust individual primer concentrations. This can be done by either increasing primer concentration of low-coverage amplicons or by decreasing the primer concentration of the high-coverage amplicons. We performed a primer concentration adjustment on the breast cancer panels, halving the concentration of primers that had an overrepresentation in sequencing coverage (Fig. 3b, shaded in orange), whilst doubling the concentration of those with low coverage (Fig. 3b, shaded in green). Together, this improved the balance of the coverage between the amplicons (Fig. 3b, as observable in the top panel through the difference between grey versus blue boxes and the barplot in the bottom panel).

##### PCR bias

A further critical post-sequencing quality control step is the assessment of PCR bias as this can affect the accuracy of estimation of the DNA methylation levels. To aid in the accurate quantitation of methylation levels, we included fully methylated and unmethylated control DNA (Zymo whole-genome amplified (WGA)) and a gradient of methylated-control DNA samples (e.g. 0%, 10%, 25%, 50%, 75%, 90%, 100%) to compare the observed versus expected levels of methylation. Here, we demonstrate calibration of DNA methylation levels using the prostate cancer DNA samples. Using a previously published formula [28, 29], we mathematically assessed PCR bias (*b*) for each of our amplicons in the prostate cancer panels (Fig. 3c (i), left panel for two example amplicons). Next, we used *b*, the bias estimate, for each amplicon in a regression (as described in Moskalev et al. [29]) to correct the observed levels of methylation of the gradient methylated-control DNA samples to closely resemble expected levels (Fig. 3c (ii), left panel). Having verified the bias correction for each amplicon, we then used the same calculated bias estimates to perform PCR bias correction of the cancer DNA samples (Fig. 3c (iii), ‘samples’) run in the same experiment as the methylated-control DNA. We observed that following correction, the multiplex data has a wider dynamic range of methylation and is more similar to matched whole-genome bisulphite methylation data from the same samples (Fig. 3c (iv), right panel). These bias plots and calculations can be performed using our recently developed *MethPanel* shinyApp [27].

##### Sensitivity

**Fig. 4:**
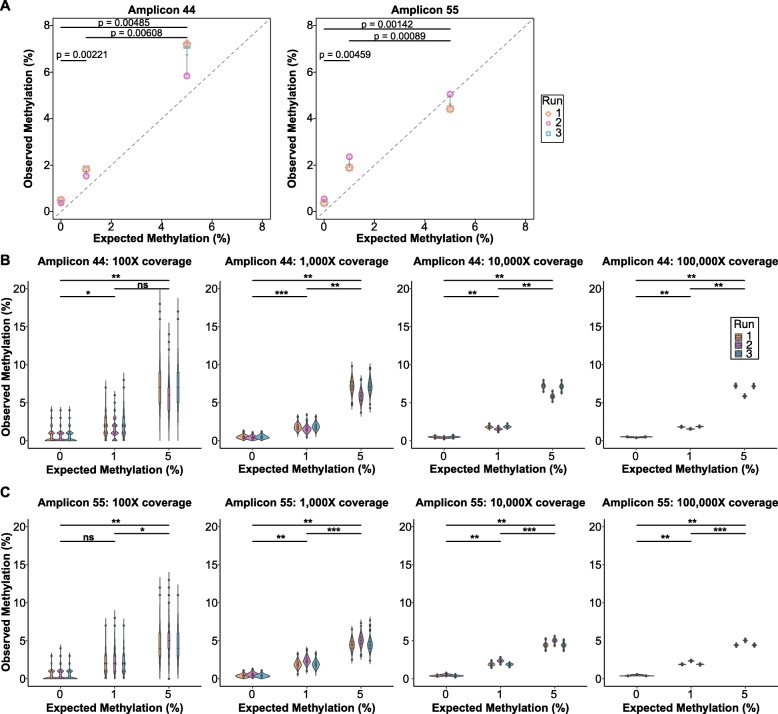
Technical sensitivity and coverage. Sensitivity of the MBPS assay was assessed using methylated-control DNA at 0%, 1% and 5% expected levels of methylation, across three separate sequencing runs. Two representative amplicons (amplicons 44 and 55) are shown. Observed methylation (%) (*y*-axis) is plotted against expected methylation (%) (*x*-axis). **a** Both representative amplicons show statistically significant detection of both 1% and 5% methylation. **b**, **c** Down-sampling sequencing coverage at 100x, 1000x, 10,000x and 100,000x shows that the sensitivity of detection of methylation improves with increased coverage. 1000x coverage and greater enables detection of 1% methylation that is statistically significant. ns = not significant (*p* ≥ 0.05), * = 0.01 ≤ *p* < 0.05, ** = 0.001 ≤ *p* < 0.01, *** = 0.0001 ≤ *p* < 0.001

Another important quality control step is to assess the sensitivity of each amplicon to determine how much sequencing coverage is required to confidently distinguish small changes in methylation levels. Utilising the fully methylated and unmethylated control DNA, we generated a gradient of methylated-control DNA samples (0%, 1% and 5%) and sequenced these across three separate sequencing runs. By comparing observed levels of DNA methylation (from sequencing, coverage > 161,335 [amplicon 44] and coverage > 244,361 [amplicon 55]) to expected levels of methylation, we assessed the technical sensitivity of the assay and found a significant difference between 0%, 1% and 5% methylation levels (Fig. 4a). By down-sampling at different sequence coverage levels, we found that the sensitivity of the assay improves with increased coverage, for example, for amplicons 44 and 55, we sensitively detected methylation differences of 1% with 1000x coverage and greater (Fig. 4a, b). Additional representative amplicons are shown in the supplementary materials (Additional file 4: Figure S3).

### Evaluation of the performance of multiplex bisulphite PCR sequencing

In the following sections, we provide evidence about the reproducibility of the method, its application to DNA from different sample types and the level of detail about DNA methylation that it can provide.

#### Methylation concordance between multiplex bisulphite PCR and whole-genome bisulphite sequencing

**Fig. 5:**
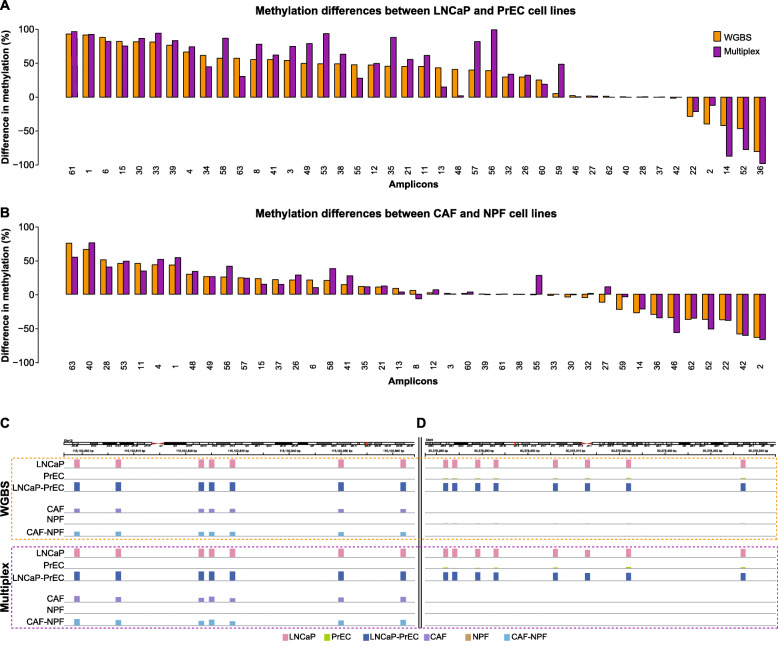
Cross-platform comparison of multiplex bisulphite PCR method and whole-genome bisulphite sequencing methylation data. Barplot shows the difference in methylation between **a** LNCaP and PrEC and **b** CAF and NPF for the prostate cancer panels, as measured by WGBS (orange bars) and MBPS (purple bars). The methylation data between the two platforms shows good concordance in determining methylation differences. **c**, **d** Representative examples of prostate DMRs corresponding to amplicon 1 (**c**) and amplicon 32 (**d**) showing WGBS and multiplex data for each cell line: LNCaP (light pink), PrEC (light green), LNCaP-PrEC (dark blue), CAF (violet), NPF (light brown) and CAF-NPF (light blue). The height of each bar represents the percentage of DNA methylation at each CpG site across the amplicon region

One of the main applications for the MBPS assay is to validate methylation changes identified from genome-wide methylation analyses. It is therefore important that the assay can provide accurate methylation data that is consistent from the discovery phase platforms. To assess this, we performed a DNA methylation comparison between WGBS and the MBPS assay data from the prostate cancer panels, for matched data from normal human prostate epithelial cells (PrEC), prostate cancer epithelial cells (LNCaP), non-malignant prostate fibroblasts (NPF) and cancer-associated fibroblasts (CAF) [24]. For the prostate cancer panels, the bias assessment led us to correct the bias of 24/63 amplicons and we used the bias-corrected data in our technical comparison. We first examined the correlation between absolute methylation values of each CpG site (158 CpG sites across 63 amplicons) as measured by the two platforms. The correlation coefficients were highly significant in each case (PrEC: *r* = 0.80, *p* < 2.2e−16; LNCaP: *r* = 0.91, *p* < 2.2e−16; NPF: *r* = 0.84, *p* < 2.2e−16; CAF: *r* = 0.95, *p* < 2.2e−16), but not completely concordant with WGBS data (Additional file 5: Figure S4A). Next, we analysed the relative methylation differences between cancer and normal sample pairs (i.e. differences between LNCaP and PrEC, and between CAF and NPF cells), with CpG methylation averaged across each amplicon. We observed improved concordance in relative methylation difference (compared to absolute methylation values) between WGBS and MBPS (Fig. 5), with highly significant correlations of LNCaP–PrEC: *r* = 0.93, *p* < 2.2e−16; CAF–NPF: *r* = 0.94, *p* < 2.2e−16 (Additional file 5: Figure S4B). This indicates that the MBPS assay is able to accurately replicate the DNA methylation differences between cell types as measured by discovery phase WGBS data (Fig. 5).

#### Performance of multiplex bisulphite PCR sequencing on FFPET DNA

**Fig. 6:**
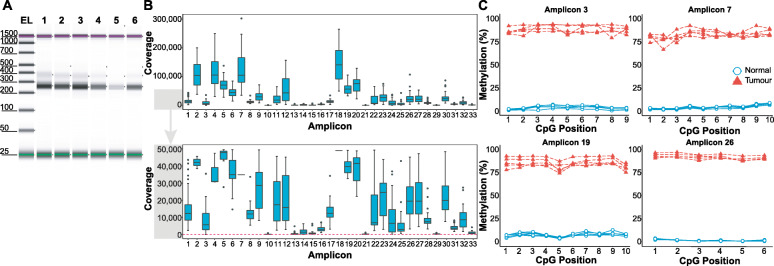
Multiplex bisulphite PCR amplicon sequencing of bisulphite-treated FFPET clinical DNA. **a** TapeStation gel showing 6 representative sequencing libraries from breast cancer FFPET-derived DNA samples are the correct sizes (~ 250 bp). EL = electronic ladder is shown. **b** Boxplot showing full coverage (top panel) across the 33 amplicons of the breast cancer panels from a sequencing run on our normal and tumour FFPET samples. Bottom panel shows the same data with a different *y*-axis scale to better show the difference between the lower coverage amplicons, with the dashed line indicating the cut-off (100 reads). **c** Line plots showing methylation data of 4 representative amplicons across 5 normal and 5 tumour clinical FFPET samples, demonstrating distinct separation between the methylation of normal and tumour samples

As clinical samples are often preserved as FFPET, we assessed the potential clinical utility of the MBPS by evaluating its performance on DNA isolated from FFPET. We performed the MBPS assay on 16 normal and 30 breast tumour FFPET DNA samples (~ 10–20 ng) using the breast cancer panels with previously optimised primer conditions (10 μM, 56 °C cycling temperature). Following PCR amplification, clean-up and library preparation, adequate amounts of sequencing libraries were produced, at the correct sizes with minimal primer dimer products (Fig. 6a; Additional file 6: Figure S5A). The libraries were then sequenced with coverage all above 100 reads (Fig. 6b). We consistently observe, across all amplicons, a clear and significant separation between lowly methylated normal and highly methylated tumour DNA (Fig. 6c), similar to differences observed in the original discovery study [25]. Together, this provides a technical validation of the MBPS assay for FFPET DNA.

#### Performance of multiplex bisulphite PCR sequencing on circulating cell-free DNA

**Fig. 7:**
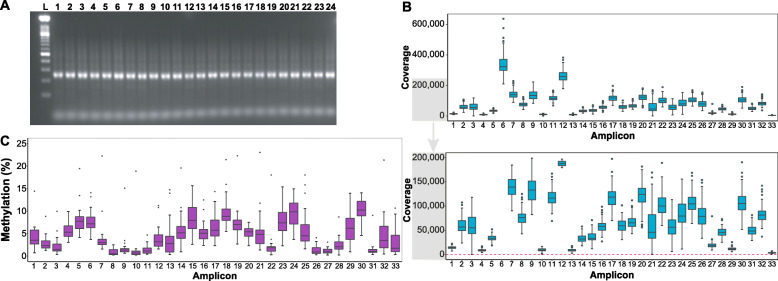
Analysis of circulating cell-free tumour DNA using multiplex PCR bisulphite sequencing. **a** Gel showing successful MBPS libraries (~ 250 bp) of 24 breast cancer-derived circulating cfDNA samples (pre-PCR cleanup). L = ladder. **b** Boxplot showing full coverage (top panel) across the 33 amplicons of the breast cancer panels from a sequencing run on the 24 cfDNA tumour samples. Bottom panel shows the same data with a different *y*-axis scale to better show the difference between the lower coverage amplicons, with the dashed line indicating the cut-off (100 reads). **c** Boxplot of the methylation values detected using the MBPS assay across 24 cfDNA samples

With the rapidly growing focus on the clinical utility of liquid biopsy monitoring, we assessed the performance of the breast cancer MBPS assay on circulating cfDNA. We used the breast cancer panels with previously optimised PCR conditions (10 μM PCR primer and 56 °C) and performed the MBPS assay on *n* = 24 tumour cfDNA samples using ~ 1–5 ng of input cfDNA. Despite the limited amount of input DNA, we observed robust PCR amplification (15 cycles PCR) of all cfDNA samples (Fig. 7a). After PCR amplification and clean-up, libraries were prepared, quantitated and sequenced on the NextSeq500 (Additional file 6: Figure S5B). The sequencing data revealed a wide range of sequencing reads across the 33 PCR amplicons, with coverage ranging from an average of 78,000 to a maximum of 638,000 reads (Fig. 7b). We were also able to measure DNA methylation levels in all amplicons in the tumour cfDNA, as shown in Fig. 7c. This data highlights the capability of the MBPS assay to detect methylation levels in liquid biopsy samples and its potential utility for monitoring epigenetic biomarkers in clinical samples.

### Use of multiplex bisulphite PCR sequencing to assess epigenetic heterogeneity

**Fig. 8:**
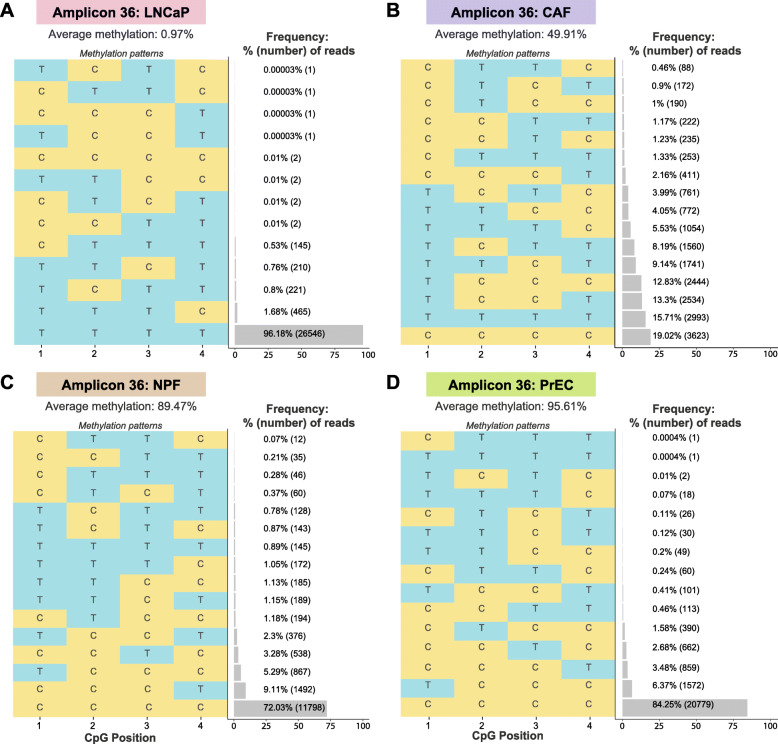
Epigenetic heterogeneity across different amplicons and samples. Matrix plot showing the read-level methylation sequencing data of each CpG dinucleotide across a candidate amplicon (amplicon 36, prostate panel) with 4 CpG sites, in each of the 4 cell types: **a** LNCaP, **b** CAF, **c** NPF and **d** PrEC. C denotes methylated cytosine; T denotes unmethylated cytosine. Barplots show the percentage frequency and number of reads of each methylation pattern

Another advantage of a targeted MBPS assay is the ability to discern the frequency of different DNA methylation patterns within each amplicon. This is informative to investigate the intra-molecular methylation heterogeneity differences between samples, for example, whether the methylation patterns in the target regions of interest indicate a difference in cellular composition between samples [30]. Figure 8 shows the different methylation patterns observed across 4 CpG sites of a representative amplicon (amplicon 36) in the prostate cancer panel in each of 4 cell types: (A) LNCaP, (B) CAF, (C) NPF and (D) PrEC. For example, Fig. 8a shows that for LNCaP, amplicon 36, we observe no methylation across all 4 CpG sites in 96.18% of reads, whereas the frequency of mosaic methylation patterns varies from 0.00003 to 1.68% of reads, giving an overall average methylation of 0.97%. In contrast, there is a large difference in average methylation levels between CAF (49.91%) and NPF cells (89.47%) for amplicon 36. Figure 8b and c show that the reduction in average methylation observed between the NPF cells and the matched CAF cells is driven by an increased frequency of different mosaic methylation patterns (seven patterns occur with > 5% frequency in the CAF), rather than the takeover of a specific clonal DNA methylation pattern.

## Discussion

There is widespread interest in DNA methylation as a molecular biomarker in disease and cancer, with several advantages that qualify DNA methylation for broad use in clinical diagnostics: (1) DNA methylation is cell-type specific, (2) it is a stable mark on DNA over cell division, (3) the patterns of methylation are faithfully retained during long-term storage as fresh-frozen or FFPET samples and (4) the methodology to assay DNA methylation biomarkers is already present in many clinical laboratories, as the assays are similar to those in use for DNA-sequence-based biomarkers. Previously, a landmark study compared all methods for DNA methylation analysis compatible with routine clinical use and concluded that targeted (locus-specific) bisulphite PCR sequencing and pyrosequencing had the ‘best all-round performance’ for biomarker development and clinical diagnosis [22]. Amplicon bisulphite sequencing has been further advanced through ‘multiplexing’ of the primers for *simultaneous* interrogation of multiple DNA methylated regions in clinical samples, a critical improvement for an assay where clinical material is very limited [23].

In this paper, we describe an updated protocol for targeted multiplex bisulphite PCR sequencing, highlight new optimisation steps to enhance its features and utility and conduct a comprehensive evaluation of its performance. We show that pre- and post-sequencing optimisation improves the performance of this MBPS assay. Pre-sequencing optimisation of PCR conditions such as temperature, primer concentration and DNA input achieves a balance between robust PCR yield and minimal primer dimer formation. Post-sequencing poorly performing individual amplicons can be rescued, either by creating a new ‘sub-panel’ or adjusting the concentrations of individual primers to equalise the coverage between the amplicons.

Methylated and unmethylated regions have different nucleotide sequence compositions after bisulphite conversion which means that, for some regions, there may be preferential amplification of either the unmethylated (T-rich) or methylated (C-rich) sequence. Amplification bias towards a particular sequence will significantly affect the accuracy of methylation quantification [28]. It is therefore important to include a gradient of quantitative methylated-control DNA samples, to detect and correct PCR bias by comparing the expected to the observed levels of methylation. We used a previously published formula [28, 29] to quantify each amplicon in the MBPS prostate cancer panels, identify PCR bias in some amplicons and correct bias prior to downstream analysis. These steps are important because PCR bias has been largely overlooked in current targeted bisulphite PCR methodologies, and so inaccurate methylation level measurements can affect the analysis and interpretation of the results.

We conducted a range of technical experiments to evaluate the performance of the updated MBPS protocol. We compared MBPS and whole-genome bisulphite sequencing data and found a significant correlation of absolute methylation values. However, there was not complete concordance. This is likely due to the low sequencing coverage of the WGBS data (genome-wide sequencing coverage > 7X for CAF and NPF and > 20X for LNCaP and PrEC, compared to an average of 68,000 reads in our prostate multiplex panel). Interestingly, we show that the correlation between MBPS and WGBS was stronger for measuring relative methylation difference between samples than absolute methylation values, suggesting that any between-platform measurement differences are uniform across all samples.

Using the methylated-control DNA samples, we also evaluated the sensitivity of the assay. We show that we can accurately detect differences in methylation between 0%, 1% and 5% methylation. Through down-sampling sequencing coverage, we can detect 1% methylation differences with as a little as 1000x coverage. Other approaches, such as methylation-specific PCR methods (MSP-PCR), can also detect low levels of methylation down to 0.1% [31, 32]; however, it is important to note that these approaches do not assess the methylation of individual CpG sites in the amplicon and are limited to single amplicons rather than the multiple regions simultaneously targeted by a multiplex assay. As well as detecting small changes in DNA methylation averaged across a population of cells, targeted MBPS also allows the assessment of heterogeneous DNA methylation patterns within cell populations. Detection of subtle changes in epigenetic profiles, for example, between normal and cancer cells, promises to reveal rare cell populations by detailing changes in different levels of cell type-specific mosaic methylation patterns [30, 33].

A key strength of the MBPS assay is the generation of high-quality next-generation sequencing data on very low-input archival and fragmented FFPET DNA (5 ng) which is important for validation and retrospective screening studies. Further, the MBPS assay can measure cfDNA methylation. This is of great clinical interest as cfDNA in human blood can serve as a liquid biopsy to provide a minimally invasive method for predictive and prognostic marker detection [34]. Levels of cfDNA are generally very low, ranging from ~ 0 to 50 ng/ml blood, and the isolated cfDNA is commonly ~ 170–300 bp, mostly corresponding to ~ 170 bp mono-nucleosomal and ~ 300 bp di-nucleosomal DNA fragments. Here, we show that minimal cfDNA (~ 1–5 ng) can generate high-quality sequencing libraries to evaluate DNA methylation, highlighting the potential utility of this approach for serial liquid biopsy monitoring of response to therapy and disease relapse.

Other notable advantages of MBPS are that it is scalable in terms of numbers of samples, easily adjustable in number of regions examined and has high reproducibility. It should be noted that another targeted bisulphite sequencing method, which uses molecular-inversion (padlock) probes, demonstrates greater multiplex scalability and is able to target thousands, rather than hundreds, of genomic regions [35, 36]. Similar to MBPS, this method can also be performed on low DNA input, such as 10–15 ng cfDNA starting material for biomarker development [36]. However, a comparison between amplicon bisulphite sequencing methods (like MBPS) and the padlock approach showed a lower percentage of reads passing quality control and lower number of mapped target regions in the padlock approach compared to the 100% success from amplicon bisulphite sequencing [22].

There are commercially available platforms for targeted methylation profiling, such as methyl-capture sequencing [37, 38] and microfluidics-based Fluid Access Array System [39]. However, both these methodologies require high-quality, high-input DNA amounts (methyl-capture seq 500 ng–3 μg; Fluid Access Array 50 ng), and thus are ill-suited to working with limited clinical samples [23]. Methyl-capture sequencing platforms offer the ability to design customizable panels to regions of clinical interest, as well as pre-designed panels (up to 5 million CpGs) [37, 38]. This extensive genomic coverage makes this method well-suited for profiling large regions of the epigenome and is thus good for biomarker discovery. However, it is not the preferred option for biomarker validation because it is less sensitive in detecting small effect sizes (5–10%) [38] and would require re-synthesis of the capture pool when adjusting the number of targets, such as for when drop-outs occur, which indicates a more time-consuming and costly technique. Furthermore, capture-based methods are unable to capture very lowly represented molecules, compared to PCR-based methods which can amplify all molecules including heterogeneously methylated molecules [40]. Thus, MBPS offers a method with higher sensitivity, cheaper costs and faster turnaround times.

One limitation of MBPS, and for the aforementioned capture- and microfluidic-based platforms, is that they all rely on the process of bisulphite conversion. This is a harsh chemical treatment that degrades and damages DNA, which can lead to the generation of libraries with low complexity and thus sequencing biases [41, 42]. This is not ideal for working with clinically derived DNA which can already be degraded and low yield. Recently, new techniques have been developed to address this problem, such as TET-assisted pyridine borane sequencing (TAPS) [43] and enzymatic methyl-sequencing (EM-seq) [44]. These methods both rely on TET enzymatic reactions to deaminate methylated cytosines. Unlike bisulphite conversion, this reaction occurs on double-stranded DNA, which preserves DNA integrity and thus allows the generation of high-quality sequencing data from low-input amounts (TAPS 1 ng gDNA/cfDNA; EM-seq 100 pg gDNA). This is highly advantageous for working with clinical material, although thus far, these promising techniques have only been applied to epigenome-wide profiling, as an alternative to WGBS, suitable for biomarker discovery. In the future, these bisulphite-free approaches could be combined with targeted multiplex methods, such as the multiplex PCR sequencing approach presented in this study, for even more sensitive and accurate biomarker validation, and thus accelerated clinical translation.

In summary, the MBPS assay can evaluate DNA methylation levels of individual CpG sites across multiple regions simultaneously including from FFPET DNA and cfDNA. Overall, the MBPS assay provides a promising approach for assessing DNA methylation in clinical samples, with potential applications in validation studies, biomarker development and clinical diagnostics, including prospective blood-based monitoring of patients.

## Material and methods

### DNA samples and extraction

DNA was extracted from LNCaP, PrEC, CAF and NPF prostate cells as described in Pidsley et al. [24]. DNA from clinical FFPET samples was extracted using the QIAamp DNA FFPET Tissue Kit (Qiagen, Cat. No. 56404), and cfDNA (Bellberry Ethics Application 2015-12-817-PRE-4) was extracted from plasma using QIAamp Circulating Nucleic Acid Kit (Qiagen, Cat. No. 55114), according to the manufacturer’s protocol. ‘Test’ DNA used was human genomic blood DNA (Roche Cat. No. 11691112001). Extracted DNA was quantified with the Qubit dsDNA HS Assay Kit (Life Technologies, USA). DNA was stored at − 20 °C until use.

### Methylated-control DNA samples

Methylated-control DNA samples were prepared by mixing 0% and 100% methylated DNA, commercially sourced from Zymo (whole-genome-amplified (WGA) non-methylated and methylated DNA, Cat. No. D5013), in the proportions needed to produce the respective methylated control. These methylated controls were included in each sequencing run to assess both PCR bias (e.g. 0%, 10%, 25%, 50%, 75%, 90%, 100%) and sensitivity (e.g. 0%, 1%, 5%). Accurate quantitation of WGA methylated and non-methylated DNA was performed by qPCR using 4–6 candidate gene regions under standard PCR conditions.

### Multiplex bisulphite PCR sequencing protocol

Figure 1 outlines the major steps in the multiplex bisulphite PCR sequencing protocol (as described below)—a more detailed version is provided in the supplementary information (Additional file 1: Figure S1) and our step-by-step protocol (Additional file 2: step-by-step protocol).

#### Primer design

Multiplex primers were designed using the custom multiplex-specific primer design software, PrimerSuite (www.primer-suite.com) [26, 45] which was adapted to use the PrimerROC software to determine the optimal PCR assay design parameters which would eliminate primer dimer artefacts when performing multiplex amplification [45]. In brief, PrimerROC was used to first determine the optimal free-energy cut-off for the multiplex assay to minimize dimer formation, which was then applied as a filter to the multiplex assay design. As PrimerRoc is now available publically (http://www.primer-dimer.com/roc/), this can be applied to any multiplex panel designed through PrimerSuite. Next, an additional, multiplex-specific DNA base-pairing heuristic was utilised to predict in silico which PCR assays need to be removed due to the likelihood that they would cause primer-primer interactions; previously, the software required users to perform each singleplex assay using quantitative PCR to determine its relative efficiency and then use the PrimerPlex module to pool assays together. PrimerSuite was then run with the following parameters: oligo melting temperature of 54 °C, sodium concentration of 50 mM and maximum of 1 CpG allowed within primers. Where there was a CpG site in the primer sequence, we substituted the cytosine with a Y/R base to limit bias. Amplicon sizes were set between 105 and 150 bp for FFPET DNA samples and between 100 and 130 bp for circulating cfDNA samples. For prostate cell lines, primers were designed for differentially methylated regions described in Pidsley et al. [24], resulting in two multiplex panels covering a total of 63 regions. For breast cancer FFPET and cfDNA samples, primers were designed for breast cancer-associated regions described in Stirzaker et al. [25], resulting in two multiplex panels covering 33 regions. Following primer design, we compared the primer sequences with dbSNP data in UCSC Genome Browser to confirm that they did not overlap any common SNPs and advise that other users of PrimerSuite do the same.

#### Bisulphite conversion

Bisulphite conversion was performed using the EZ DNA Methylation-Lightning Kit (Zymo Research, USA, Cat. No. D5030 and D5033) according to the manufacturer’s instructions. Based on the available material, as well as minimum DNA input needed, approximately 1–100 ng of each sample was bisulphite converted. For FFPET DNA samples, 2 μl of 10x bisulphite DNA lysis buffer (10 mg/ml tRNA (20 μg/ml final), 20 mg/ml Proteinase K (2.8 mg/ml final), 20% SDS (10% final)) was added to 18 μl of the starting DNA material and incubated at 55 °C for 1 h, before proceeding to the kit. For cfDNA samples, approximately 1–5 ng of each sample was directly bisulphite converted using the EZ DNA Methylation-Lightning Kit (Zymo Research, USA, Cat. No. D5030 and D5033) according to the manufacturer’s instructions.

#### Optimisation of multiplex primers

Designed primers (ordered from Integrated DNA Technologies) were first individually diluted to 100 μM with ultrapure water according to instructions given. Equal volumes of forward and reverse primers, per primer pair, were combined to dilute to a final concentration of 20 μM each. We first ran singleplex PCRs with all individual primer pairs on test DNA and no-template control. PCR reactions for the amplification of bisulphite-converted DNA had the following components (final volume = 10 μl): 5X Promega GoTaq Flexi Buffer (2 μl, Cat. No. M891A), CES 5X (1 μl, refer to Ralser et al. 2006 for CES recipe [46]), 25 mM MgCl_2_ (2 μl), 1 M TMAC (0.15 μl, tetramethylammonium chloride solution, Sigma, Cat. No. T3411-500ML), dNTPs (0.1 μl, 10 mM each), primers (1 μl, forward and reverse at 2 μM each), 5 U/μl Promega GoTaq Hot Start Polymerase (0.04 μl, Cat. No. M500B) and DNA (1 μl, 10 ng/μl for optimisation PCRs). The PCR cycling conditions were 94 °C, 7 min; 40 cycles (94 °C, 20 s; 55–57 °C, 30 s; 72 °C, 2 min); 72 °C, 5 min; 4 °C hold. PCR products were run on a 2% agarose (with TAE buffer) gel electrophoresis to check the specificity of each individual primer pair and ensure bands at the correct size (according to PrimerSuite design) and minimal primer dimer formation. Should any individual PCR fail (very faint or no bands), we recommend that these primers are discarded and new primers designed for these regions.

Following singleplex PCR, equal amount of each primer pair was then pooled into their respective panels, based on the results of the PrimerSuite software. Multiplex PCR reactions (see Additional file 2: section 2.3.3) for reaction mix and cycling conditions were performed at varying cycling temperatures (e.g. 55–57 °C) and primer concentrations (e.g. 20 μM, 10 μM, 5 μM, 2.5 μM) to optimise these components. The optimal temperature and primer concentration yielded the most product with minimal primer dimers, as visualized by gel electrophoresis. These optimal conditions were used in all subsequent steps. As low DNA yield may result from working with patient clinical samples, additional multiplex PCRs were performed with varying levels of DNA input (e.g. 10 ng, 5 ng, 2.5 ng, 1.25 ng, 0.625 ng) to assess the minimal amount of DNA that these multiplex primers required for optimal amplification (see Additional file 2: section 2.3.4).

#### Phosphorylation of primers and multiplex bisulphite PCRs

Following optimisation, the multiplex pooled primers were phosphorylated to assist in the ligation of Illumina indexing adapters below. This was done using an in-house recipe (Additional file 2: section 2.4.1). Starting with twice the optimal concentration of pooled primers (such that for a final concentration of 10 μM, start with 20 μM of pooled primers), the following mix was made (final volume = 50 μl): pooled primers (37.5 μl), 10X NEB DNA ligase buffer (2 μl, Cat. No. B0202S), T4 polynucleotide kinase (2 μl, Cat. No. M0201L), 10 mM ATP (5 μl, Cat. No. P0756S) and 1 M DTT (0.25 μl, Thermo Fisher, Cat No. P2325). This reaction was performed at 37 °C for an hour. The reaction was then topped up with the following (final volume = 25 μl): 10X NEB ligase buffer (2.5 μl), T4 polynucleotide kinase (1 μl), 10 mM ATP (2.5 μl) and 1 M DTT (0.25 μl). The reaction was performed for another hour at 37 °C. The multiplex pooled primers were then at the optimal concentration (e.g. 10 μM).

Using these phosphorylated pooled primers, multiplex bisulphite PCRs were performed on bisulphite-treated patient DNA, cell line DNA and methylated-controls, in triplicate under optimised multiplex PCR conditions. The PCRs were performed in 15 μl reactions with following components: 5X Promega GoTaq Flexi Buffer (3 μl, Cat. No. M890A), CES 5X (1.5 μl), 25 mM MgCl_2_ (3 μl), 1 M TMAC (0.225 μl), dNTPs (0.15 μl, 10 mM each), phosphorylated primers (3 μl, at optimal concentration e.g. 10 μM), 5 U/μl Promega GoTaq Hot Start (0.06 μl) and DNA (2 μl). The PCR cycling conditions were similar to above, using the optimal cycling temperature as determined during the optimisation steps, and only 28 cycles were performed here. The optimal DNA final concentration is 0.5–1 ng/μl, subject to availability of DNA material (see optimisation PCRs for determination of minimum DNA input required). The triplicate PCRs were pooled and PCR cleanup performed using Agencourt AMPure XP beads (Cat. No. A63881) at a 1:1.6 (up to 1:2) ratio (see Additional file 2: section 2.4.3 for clean-up procedure).

#### Library preparation

Following PCR clean-up, TruSeq Dual Index Adaptors (Illumina, Part No. 15032317) were ligated to each sample. To each cleaned PCR product, 1:20 dilution (0.75 μM) of adaptors and ligation mastermix (see Additional file 2: section 2.5.1 for recipe) were added accordingly (final volume = 11.2 μl): cleaned PCR product (7 μl), 0.75 μM adaptors (1 μl) and ligation mastermix (3.2 μl), then incubated at 37 °C for 30 min. A further round of 8–12 cycles of PCR (see above for cycling conditions) was performed to amplify the libraries and incorporate the Illumina sequencing primers (P5 & P7, TruSeq DNA Library Prep Kit HT). The reaction mix was as follows (final volume = 40 μl): 5X Promega GoTaq Flexi Buffer (8 μl), 25 mM MgCl_2_ (9.6 μl), dNTPs (0.4 μl, 10 mM each), P5 and P7 primers (4 μl, 10 μM), 5 U/μl Promega GoTaq Hot Start Polymerase (0.25 μl) and ligated DNA (5 μl). The libraries were then purified using AMPure XP beads at a 1:1 ratio.

Following library purification, each library was quantified using the Qubit dsDNA HS Assay Kit (Life Technologies, USA). Representative libraries, including lowest and highest concentration libraries, were checked on TapeStation (D1000, Cat. No. 5067-5602 and 5067-5582). One microliter of library mix with 3 μl buffer per library was run on the TapeStation machine to check library size (~ 250 bp based on amplicon size plus sequencing adaptors) and purity. The individual libraries were then pooled at equal amounts (for each sequencing run—96 samples) and the pooled libraries run on the TapeStation. If primer dimer bands (< 200 bp) were observed, a second clean-up (1:1 AMPure XP beads) was performed, and the quantification steps above were repeated. The pooled libraries were then diluted to 10–20 nM according to further Qubit quantification. KAPA qPCR (KAPA Library Quantification Kit (ABI Prism), Roche, Cat. No. 07960204001) was then performed on the pooled library, according to manufacturer’s instructions. The pooled library was then diluted to 10 nM according to the KAPA qPCR results, ready for sequencing.

#### Sequencing

Sequencing of the LNCaP, PrEC, CAF and NPF prostate cells was performed on the Illumina MiSeq™ sequencer (Illumina, CA, USA). Sequencing of the breast cancer FFPET DNA and cfDNA samples was performed on the Illumina NextSeq™ sequencer (Illumina, CA, USA). Methylated controls were included in each sequencing run. Sample preparation for sequencing on these machines was performed according to Illumina’s instructions, with library concentration and addition of PhiX Control v3 (Illumina, FC-110-3001) optimised for the individual machines.

### Data analysis/bioinformatics

#### Processing of multiplex sequencing data

We used the *MethPanel* workflow [27] to preprocess and align reads from multiplex bisulphite sequencing to pre-defined regions of the reference genome hg19 build (defined using the genomic co-ordinates for each amplicon from the output from the PrimerSuite software). Specifically, FASTQ files were trimmed to produce high-quality reads with base quality ≥ 30 and read length ≥ 20 bp and to clip 1 bp from both reads (https://github.com/FelixKrueger/TrimGalore). Non-conversion of non-CpG cytosines was used to estimate bisulphite conversion rate (typically > 99.4%). *Bismark* version 0.22.3 [47] was used to map these trimmed reads to the pre-defined reference genome, allowing 1 non-bisulphite mismatch per read, with all other parameters kept to their default values. Sequencing metrics for all runs in this study are summarized in Additional file 7: Supplementary Table 1. For each bam file produced by *Bismark*, *MethPanel* [27] was used to perform calculation of DNA methylation levels and merge all samples into a single table. Further quality control was performed to remove amplicons and samples with < 100X coverage from the methylation table. All downstream analysis and data visualization were conducted using *MethPanel* or custom scripts in R (version ≥ 3.2.2) [48]. For results where we present a single methylation value for an amplicon, this value was derived by taking the mean methylation of all CpG sites in an amplicon for each sample.

#### Whole-genome bisulphite sequencing data

For CAF and NPF methylation, we used in-house WGBS data that was generated and processed as previously described [24]. All raw and processed data are publically available at NCBI Gene Expression Omnibus (GEO) (www.ncbi.nlm.nih.gov/geo) under accession number GSE86260, sample names: WGBS CAF2 and WGBS NPF2.

For LNCaP and PrEC methylation, we used in-house WGBS data that was generated and processed as previously described [19]. All raw and processed data are publically available at NCBI GEO (www.ncbi.nlm.nih.gov/geo) under accession number GSE86832.

To allow comparison of WGBS data with multiplex data, we created a bed-formatted file of the 158 CpG sites covered by the multiplex panel and applied the ‘getMeth’ function in the bsseq package in R to extract WGBS data at these sites.

## Supplementary information

**Additional file 1: Figure S1.** Detailed flow diagram of multiplex PCR bisulphite sequencing assay.

**Additional file 2:** Step-by-step protocol (Word Document)

**Additional file 3: Figure S2.** Pre-sequencing optimisation of multiplex PCR primers - Prostate Cancer panels (PDF). (A) PCR products of singleplex amplification of 63 individual primer pairs from the prostate cancer panels run on 2% agarose gel. The gel shows the specificity of all the primer pairs and PCR products of the correct size (105-150 bp) with minimal primer dimer formation. (-) no template; (+) bisulphite-treated test DNA template (10ng), (L) 100 bp DNA Ladder. (B) Singleplex primers were pooled into their respective multiplex panels and the success of multiplex PCR reactions is shown at different primer concentrations (20 μM, 10 μM, 5 μM and 1 μM) and at an annealing temperature of 56 °C. (C) PCR products from the multiplex panel testing DNA input amounts from 10 ng, 5 ng, 2.5 ng, 1.25 ng of bisulphite-treated control DNA; (+) test DNA; (-) no template control.

**Additional file 4: Figure S3.** Technical Sensitivity and Coverage (PDF). Sensitivity of the multiplex PCR bisulphite sequencing assay was assessed using methylated-control DNA at 0%, 1% and 5% expected levels of methylation, across three separate sequencing runs. Three representative amplicons are shown. Observed methylation (%) (y-axis) is plotted against expected methylation (%) (x-axis). Down-sampling sequencing coverage at 100x, 1,000x, 10,000x and 100,000x shows that the sensitivity of detection of methylation improves with increased coverage.

**Additional file 5: Figure S4.** Platform comparison of MBPS and WGBS methylation data (PDF). (A) Correlation plots of absolute methylation levels of each CpG in the prostate cancer panels, as measured by multiplex bisulphite PCR (y-axis) and WGBS (x-axis), across each of the four cells (LNCaP, PrEC, CAF, NPF). (B) Correlation plots of relative methylation differences (LNCaP minus PrEC; CAF minus NPF cells) as measured by MBPS (y-axis) and WGBS (x-axis). Pearson’s correlation test was used to derive the correlation coefficient (r) and p-value (p).

**Additional file 6: Figure S5. **MBPS of bisulphite-treated FFPET clinical DNA (PDF). Tape Station electropherogram showing representative sequencing libraries from breast cancer (A) FFPET-derived DNA samples and (B) circulating cell-free DNA samples. The grey peak at ~250 bp represents the library (amplicon + sequencing adaptors), with peaks observed < 200 bp representing residual primer dimers.

**Additional file 7: Table S1.**

## Data Availability

The datasets generated and analysed during the current study are available from the corresponding authors on reasonable request. The WGBS data (raw and processed) analysed during this study is publically available at NCBI GEO (www.ncbi.nlm.nih.gov/geo) under accession numbers GSE86260 (CAF and NPF) and GSE86832 (LNCaP and PrEC).

## Abbreviations

CAF: Cancer-associated fibroblasts
cfDNA: Circulating cell-free DNA
DMRs: Differentially methylated regions
FFPET: Formalin-fixed paraffin-embedded tissue
LNCaP: Prostate cancer epithelial cell line
MBPS: Multiplex bisulphite PCR sequencing
NPF: Non-malignant prostate fibroblasts
PrEC: Normal human prostate epithelial cells
WGA: Whole-genome amplified
WGBS: Whole-genome bisulphite sequencing

## References

[1] Bird AP (1986). CpG-rich islands and the function of DNA methylation. Nature..

[2] Jones PA (2012). Functions of DNA methylation: islands, start sites, gene bodies and beyond. Nat Rev Genet..

[3] Feil R, Fraga MF (2012). Epigenetics and the environment: emerging patterns and implications. Nat Rev Genet.

[4] Martin EM, Fry RC (2018). Environmental influences on the epigenome: exposure- associated DNA methylation in human populations. Annu Rev Public Health.

[5] Feinberg AP (2007). Phenotypic plasticity and the epigenetics of human disease. Nature..

[6] Baylin SB, Jones PA (2011). A decade of exploring the cancer epigenome - biological and translational implications. Nat Rev Cancer.

[7] Jones PA, Baylin SB (2007). The epigenomics of cancer. Cell..

[8] Baylin SB, Jones PA. Epigenetic determinants of cancer. Cold Spring Harb Perspect Biol. 2016;8(9). 10.1101/cshperspect.a019505 PMC5008069 27194046

[9] Cancer Genome Atlas N (2012). Comprehensive molecular portraits of human breast tumours. Nature..

[10] Cancer Genome Atlas Research N (2011). Integrated genomic analyses of ovarian carcinoma. Nature..

[11] Hudson TJ, Anderson W, Artez A, Barker AD, Bell C, International Cancer Genome C (2010). International network of cancer genome projects. Nature..

[12] Kristensen LS, Hansen LL (2009). PCR-based methods for detecting single-locus DNA methylation biomarkers in cancer diagnostics, prognostics, and response to treatment. Clin Chem.

[13] Mikeska T, Bock C, Do H, Dobrovic A (2012). DNA methylation biomarkers in cancer: progress towards clinical implementation. Expert Rev Mol Diagn.

[14] Umer M, Herceg Z (2013). Deciphering the epigenetic code: an overview of DNA methylation analysis methods. Antioxid Redox Signal.

[15] Lister R, Pelizzola M, Dowen RH, Hawkins RD, Hon G, Tonti-Filippini J (2009). Human DNA methylomes at base resolution show widespread epigenomic differences. Nature..

[16] Clark SJ, Harrison J, Paul CL, Frommer M (1994). High sensitivity mapping of methylated cytosines. Nucleic Acids Res.

[17] Lister R, O'Malley RC, Tonti-Filippini J, Gregory BD, Berry CC, Millar AH (2008). Highly integrated single-base resolution maps of the epigenome in Arabidopsis. Cell..

[18] Nair SS, Luu PL, Qu W, Maddugoda M, Huschtscha L, Reddel R (2018). Guidelines for whole genome bisulphite sequencing of intact and FFPET DNA on the Illumina HiSeq X ten. Epigenetics Chromatin.

[19] Pidsley R, Zotenko E, Peters TJ, Lawrence MG, Risbridger GP, Molloy P (2016). Critical evaluation of the Illumina MethylationEPIC BeadChip microarray for whole-genome DNA methylation profiling. Genome Biol.

[20] Bibikova M, Le J, Barnes B, Saedinia-Melnyk S, Zhou L, Shen R (2009). Genome-wide DNA methylation profiling using Infinium(R) assay. Epigenomics..

[21] Peters TJ, Buckley MJ, Statham AL, Pidsley R, Samaras K, Lord RV (2015). De novo identification of differentially methylated regions in the human genome. Epigenetics Chromatin.

[22] BLUEPRINT consortium, Bock C, Halbritter F, Carmona FJ, Tierling S, Datlinger P, Assenov Y, Berdasco M, et al. Quantitative comparison of DNA methylation assays for biomarker development and clinical applications. Nat Biotechnol. 2016;34(7):726–37. 10.1038/nbt.3605 27347756

[23] Korbie D, Lin E, Wall D, Nair SS, Stirzaker C, Clark SJ (2015). Multiplex bisulfite PCR resequencing of clinical FFPE DNA. Clin Epigenetics.

[24] Pidsley R, Lawrence MG, Zotenko E, Niranjan B, Statham A, Song J (2018). Enduring epigenetic landmarks define the cancer microenvironment. Genome Res.

[25] Stirzaker C, Zotenko E, Song JZ, Qu W, Nair SS, Locke WJ (2015). Methylome sequencing in triple-negative breast cancer reveals distinct methylation clusters with prognostic value. Nat Commun.

[26] Lu J, Johnston A, Berichon P, Ru KL, Korbie D, Trau M (2017). PrimerSuite: a high-throughput web-based primer design program for multiplex bisulfite PCR. Sci Rep.

[27] Luu PL, Ong P-T, Loc TTH, Lam D, Pidsley R, Stirzaker C, et al. MethPanel: a parallel pipeline and interactive analysis tool for multiplex bisulphite PCR sequencing to assess DNA methylation biomarker panels for disease detection. BioRxiv. 2020. 10.1093/bioinformatics/btaa1060 PMC8352503 33367555

[28] Warnecke PM, Stirzaker C, Melki JR, Millar DS, Paul CL, Clark SJ (1997). Detection and measurement of PCR bias in quantitative methylation analysis of bisulphite-treated DNA. Nucleic Acids Res.

[29] Moskalev EA, Zavgorodnij MG, Majorova SP, Vorobjev IA, Jandaghi P, Bure IV (2011). Correction of PCR-bias in quantitative DNA methylation studies by means of cubic polynomial regression. Nucleic Acids Res.

[30] Landan G, Cohen NM, Mukamel Z, Bar A, Molchadsky A, Brosh R (2012). Epigenetic polymorphism and the stochastic formation of differentially methylated regions in normal and cancerous tissues. Nat Genet.

[31] Cottrell SE, Laird PW (2003). Sensitive detection of DNA methylation. Ann N Y Acad Sci.

[32] Rand KN, Ho T, Qu W, Mitchell SM, White R, Clark SJ (2005). Headloop suppression PCR and its application to selective amplification of methylated DNA sequences. Nucleic Acids Res.

[33] Barrett JE, Feber A, Herrero J, Tanic M, Wilson GA, Swanton C (2017). Quantification of tumour evolution and heterogeneity via Bayesian epiallele detection. BMC Bioinformatics.

[34] Pidsley R, Stirzaker, C. Cancer methylation biomarkers in circulating cell-free DNA. Hesson L. PA, editor. Singapore: Springer; 2019. 217-45 p.

[35] Diep D, Plongthongkum N, Gore A, Fung HL, Shoemaker R, Zhang K (2012). Library-free methylation sequencing with bisulfite padlock probes. Nat Methods.

[36] Xu RH, Wei W, Krawczyk M, Wang W, Luo H, Flagg K (2017). Circulating tumour DNA methylation markers for diagnosis and prognosis of hepatocellular carcinoma. Nat Mater.

[37] Allum F, Shao X, Guenard F, Simon MM, Busche S, Caron M (2015). Characterization of functional methylomes by next-generation capture sequencing identifies novel disease-associated variants. Nat Commun.

[38] Teh AL, Pan H, Lin X, Lim YI, Patro CP, Cheong CY (2016). Comparison of methyl-capture sequencing vs. Infinium 450 K methylation array for methylome analysis in clinical samples. Epigenetics..

[39] Bourgon R, Lu S, Yan Y, Lackner MR, Wang W, Weigman V (2014). High-throughput detection of clinically relevant mutations in archived tumor samples by multiplexed PCR and next-generation sequencing. Clin Cancer Res.

[40] Jiang J, Wolters JE, van Breda SG, Kleinjans JC, de Kok TM (2015). Development of novel tools for the in vitro investigation of drug-induced liver injury. Expert Opin Drug Metab Toxicol.

[41] Tanaka K, Okamoto A (2007). Degradation of DNA by bisulfite treatment. Bioorg Med Chem Lett.

[42] Olova N, Krueger F, Andrews S, Oxley D, Berrens RV, Branco MR (2018). Comparison of whole-genome bisulfite sequencing library preparation strategies identifies sources of biases affecting DNA methylation data. Genome Biol.

[43] Liu Y, Siejka-Zielinska P, Velikova G, Bi Y, Yuan F, Tomkova M (2019). Bisulfite-free direct detection of 5-methylcytosine and 5-hydroxymethylcytosine at base resolution. Nat Biotechnol.

[44] Vaisvila RP, V.K.C.; Sun, Z.; Langhorst, B.W.; Saleh, L.; Guan, S.; Dai, N.; Campbell, M.A.; Sexton, B.S; Marks, K.; Samaranayake, M.; Samuelson, J.C.; Church, H.E.; Tamanaha, E.; Corrêa Jr., I.R.; Pradhan, S.; Dimalanta, E.T.; Evans Jr., T.C.; Williams, L.; Davis, T.B. EM-seq: detection of DNA methylation at single base resolution from picograms of DNA. bioRxiv. 2019.

[45] Johnston AD, Lu J, Ru KL, Korbie D, Trau M (2019). PrimerROC: accurate condition-independent dimer prediction using ROC analysis. Sci Rep.

[46] Ralser M, Querfurth R, Warnatz HJ, Lehrach H, Yaspo ML, Krobitsch S (2006). An efficient and economic enhancer mix for PCR. Biochem Biophys Res Commun.

[47] Krueger F, Andrews SR (2011). Bismark: a flexible aligner and methylation caller for bisulfite-Seq applications. Bioinformatics..

[48] R Core Team: a language and environment for statistical computing. Vienna, Austria: R Foundation for Statistical Computing; 2013.

