# Supplementary figures and images for: Comprehensive evaluation of targeted multiplex bisulphite PCR sequencing for validation of DNA methylation biomarker panels
Source: Clin Epigenetics. 2020 Jun 22;12:90. doi: 10.1186/s13148-020-00880-y (PMC7310104; doi:10.1186/s13148-020-00880-y)

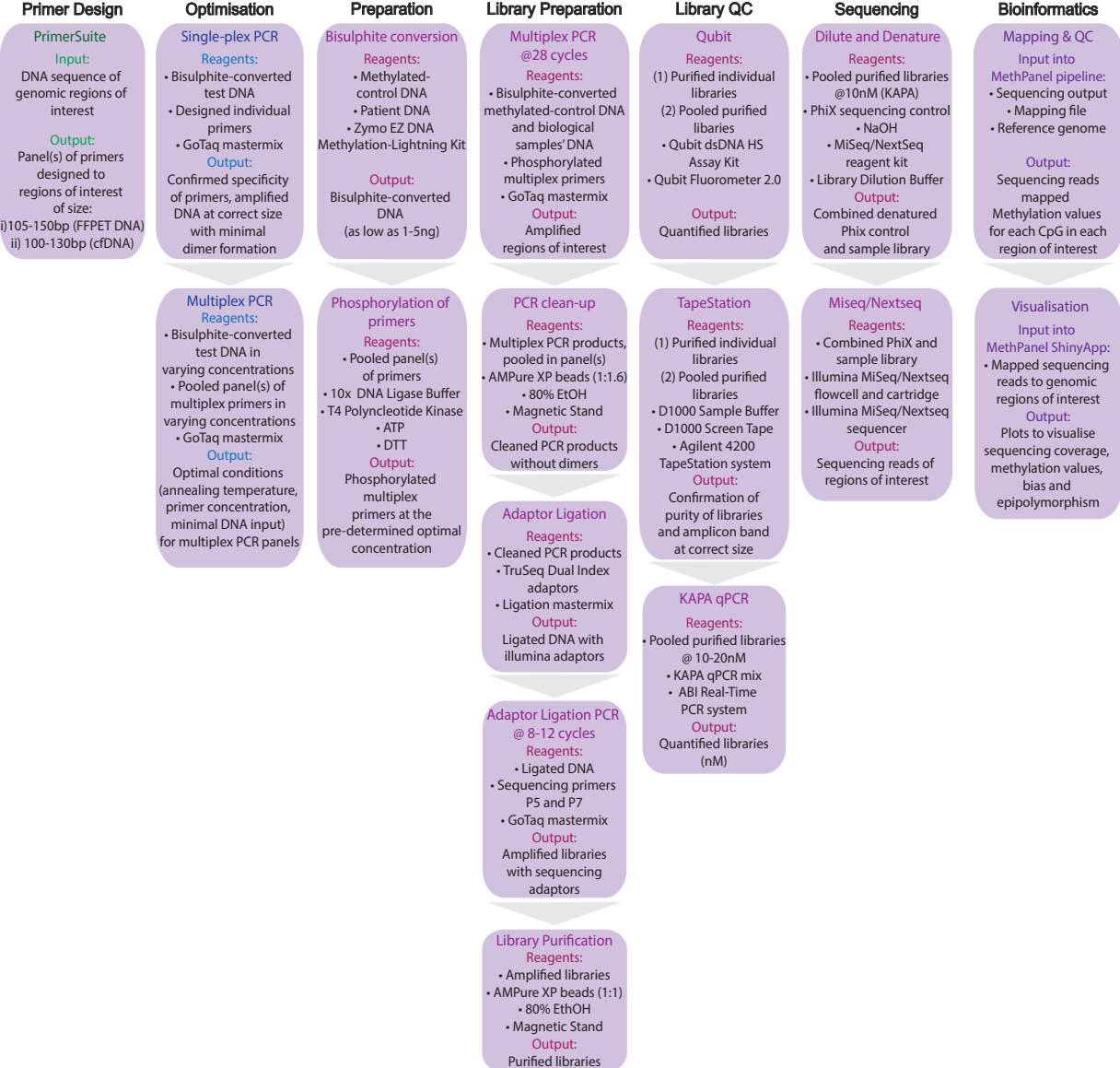

**Figure S1. Detailed flow diagram of multiplex PCR bisulphite sequencing assay**

Supplement: Supplementary file 1 — Additional file 1: Figure S1. Detailed flow diagram of multiplex PCR bisulphite sequencing assay. [file 13148_2020_880_MOESM1_ESM.pdf]
