# Supplementary material for: Comprehensive evaluation of targeted multiplex bisulphite PCR sequencing for validation of DNA methylation biomarker panels
Source: Clin Epigenetics. 2020 Jun 22;12:90. doi: 10.1186/s13148-020-00880-y (PMC7310104; doi:10.1186/s13148-020-00880-y)
Supplement: Supplementary file 2 — Additional file 2: Step-by-step protocol (Word Document) [file 13148_2020_880_MOESM2_ESM.docx]

**PROTOCOL**

**1. Materials**

***1.1 Bisulphite treatment***

1. EZ DNA Methylation-Lightning Kit (Zymo Research; Cat. No. D5030 or D5033)

2. For FFPET derived material: 10X DNA lysis buffer [10mg/ml tRNA (20ug/ml final concentration), 20mg/ml Proteinase K (2.8mg/ml final), 20% SDS (10% final)]

3. Heatblock at 55°C

4. Thermocycler

***1.2 Primer optimisation & Multiplex PCRs***

1. 5U/μl GoTaq Hot Start Polymerase (Cat. No. M500B)

2. 5X Green GoTaq Flexi Buffer (Cat. No. M891A) – for Optimisation PCRs only

3. 5X Colorless GoTaq Flexi Buffer (Cat. No. M890A)

4. 5X CES (Combinatorial Enhancer Solution) Buffer (refer to Ralser et al., 2006 for recipe)

5. 25mM MgCl_2_

6. 1M TMAC (Tetramethylammonium Chloride Solution, Sigma, Cat. No. T3411-500ML)

7. 10mM dNTP mix

8. Thermocycler

9. 2% Agarose Gel

10. TAE Buffer

11. Gel electrophoresis machine

***1.3 Phosphorylation of primers***

1. 10X DNA Ligase Buffer (NEB, Cat. No. B0202S)

2. T4 Polynucleotide Kinase (NEB, Cat. No. M0201L)

3. 10mM ATP (NEB, Cat. No. P0756S)

4. 1M DTT (Thermo Fisher, Cat. No. P2325)

5. Heatblock at 37°C

***1.4 Library Preparation***

1. Illumina library preparation is performed using the TruSeq DNA Library Prep Kit HT, 96 samples:

a. Dual Index Adaptors (part # 15032317)

b. Sequencing primers P5 & P7

c. Resuspension Buffer (part # 15026770)

2. Ultra II Ligase MasterMix (NEB, Cat. No. 37648AA)

3. Ligation Enhancer (NEB, Cat. No. E7374AA)

3. 5U/μl GoTaq Hot Start Polymerase (Cat. No. M500B)

4. 5X Colorless GoTaq Flexi Buffer (Cat. No. M890A)

5. 25mM MgCL_2_

6. 10mM dNTP mix

7. AMPure XP beads

8. 80% Ethanol (EtOH), freshly prepared

9. Magnetic Stand (Invitrogen)

10. Ultra Pure Water

11. Thermocycler

***1.5 Library Quantification***

1. Qubit dsDNA HS Assay Kit (Life Technologies, Cat. No. Q32854)

2. Qubit assay tubes (Life Technologies, Cat. No. Q32856)

3. Qubit 2.0 Fluorometer (Life Technologies, Cat. No. Q32866)

4. D1000 ScreenTape (Agilent Technologies, Cat. No. 5067-5582)

5. D1000 Sample Buffer (Agilent Technologies, Cat. No. 5067-5602)

6. Agilent 4200 TapeStation system

7. KAPA Library Quantification Kit (Illumina), ABI Prism qPCR Mix (Roche, Cat. No. 07960204001)

8. ABI 7900 Real-Time PCR system

9. Library Dilution Buffer (10mM Tris-Hcl pH 8.0, 0.05% Tween 20)

***1.6 Sequencing***

1. MiSeq^TM^ sequencer or NextSeq^TM^ sequencer (Illumina)

2. Illumina Miseq Reagent Kit v2 (300-cycles; Cat. No. MS102-2002) or NextSeq 500/550 High Output Kit v2 (300 cycles)

3. PhiX Sequencing Control v3 (Illumina, FC-110-3001)

4. Library Dilution Buffer

5. 0.2N NaOH

***1.7 Data analysis***

1. MethPanel pipeline (1)

2. ShinyApp (https://github.com/thinhong/MethPanel)

**2. METHODS**

**2.1 Primer Design Considerations**

To design primers that can be optimally co-amplified together in one PCR reaction, we recommend utilising the multiplex-specific primer design software PrimerSuite (2, 3):

1. Recommended parameters:
   1. Oligo melting temperature: 54°C
   2. Sodium concentration: 50Mm
   3. Maximum CpGs allowed within primers: 1
2. For formalin-fixed paraffin-embedded (FFPET) derived DNA, aim for designing amplicons between 105-150bp
3. For circulating cell-free DNA (cfDNA), aim for designing amplicons between 100-130bp

**2.2 Bisulphite treatment**

DNA samples are treated with sodium bisulphite to convert unmethylated cytosine to thymine, whilst methylated cytosines are protected from bisulphite conversion. We follow the procedure as provided by the manufacturer of Zymo Research EZ DNA Methylation-Lightning^TM^ Kit. For FFPET-derived DNA only, there is an additional preparation step before bisulphite treatment to improve the efficiency of bisulphite conversion treatment. Based on the available material, around 1-100ng of each sample was bisulphite treated.

1. For **FFPET-derived DNA only**: Add 2μl of 10x bisulphite DNA lysis buffer (see above for recipe) to 18μl of the starting DNA material. Incubate at 55°C for 1 hour.

***SAFE STOPPING POINT:*** *If you do not plan to proceed immediately, store your bisulphite treated DNA at -20°C (for up to 4 weeks – bisulphite treated DNA is not suitable for longer storage periods).*

**2.3 Optimisation of Multiplex Primers**

Optimisation PCRs should be run for every newly designed set of multiplex primers. For optimisation PCRs only, we perform the reaction for 35-40 cycles to ensure the resulting product can be visualised on agarose gel electrophoresis.

**2.3.1 Primer pair pooling and dilution**

1. Upon receiving custom designed primers, first dilute forward and reverse primers individually to 100μM with Ultra Pure Water according to the instructions given by the oligo manufacturer.

2. Combine equal volumes of forward and reverse primers, per primer pair, and dilute to a final concentration of 20μM each primer pair.

**2.3.2 Singleplex optimisation PCR**

This singleplex optimisation PCR tests that each custom designed primer pair can precisely amplify bisulphite-converted DNA. This is conducted on test DNA and includes no-template controls.

1. Make up 5X CES Buffer (4) and keep on ice. (Or remove previously made CES buffer from -20°C and thaw at room temperature, place on ice).
2. Remove 5X Green GoTaq Flexi Buffer, 25mM MgCl_2_, 10mM dNTP Mix from -20°C and thaw at room temperature. When thawed, briefly centrifuge and keep these tubes on ice until use. 1M TMAC should be kept at room temperature.
3. Remove your custom primers and test DNA from -20°C and thaw at room temperature, place on ice.
4. For each set of primer pair (i.e. amplicon), prepare the following reaction mix on ice in a 200 μl thin wall PCR tube, or 96-well plate:

| **Reagent** | **Volume (μl)** |
| --- | --- |
| Ultra Pure Water | 2.71 |
| 5X Green GoTaq Flexi Buffer | 2 |
| 25mM MgCl_2_ | 2 |
| 10mM dNTP Mix | 0.1 |
| 1M TMAC | 0.15 |
| 5X CES Buffer | 1 |
| Primers (at **2μM each**) | 1 |
| Test DNA (10ng/μl) | 1 |
| 5U/μl GoTaq Hot Start Polymerase | 0.04 |
| **Total Volume** | **10μl** |

1. Include, for each primer set, a negative template control (replace DNA with Ultra Pure Water in the reaction mix)
2. Vortex the reaction mix briefly to mix thoroughly, and briefly centrifuge to ensure liquid is at the bottom of the tubes/plate.
3. Perform PCR amplification using the following PCR cycling conditions with a heated lid:
   1. 94°C for 7 minutes
   2. 94°C for 20 seconds
   3. 55-57°C for 30 seconds
   4. 72°C for 2 minutes
   5. Repeat steps b-d for a total of 40 cycles
   6. 72°C for 5 minutes
   7. Hold at 4°C
4. Loading all 10μl, visualise the PCR products on a 2% agarose gel (with TAE buffer), to ensure bands for each primer pair are at the correct size (based on PrimerSuite) with minimal primer dimer formation.
5. If any individual PCRs fail, we recommend that these primers are discarded and new primers designed for these regions.

*Note: You may wish to perform optimisation of annealing temperature at this singleplex stage rather than at the multiplex stage. If so, perform a gradient PCR for each individual primer pair, varying the annealing temperature from ~55°C to 57°C).*

**2.3.3 Multiplex PCR optimisation: Primer Concentration, Annealing Temperature**

The first optimisation step in Multiplex PCR format combines optimisation of both primer concentration (e.g. 20, 10, 5, 2.5, 1 μM) and annealing cycling temperature (e.g. 55°C, 56°C, 57°C).

1. Pool equal amount of each primer pair (i.e. amplicon) together into their respective panels in 0.5ml tubes, as per the primer design software.
2. For **each panel of pooled primers**, prepare the following reaction mix with different primer concentrations on ice in a 200 μl thin wall PCR tube, or 96-well plate:

| **Reagent** | **Volume (μl)** |
| --- | --- |
| Ultra Pure Water | 1.71 |
| 5X Green GoTaq Flexi Buffer | 2 |
| 25mM MgCl_2_ | 2 |
| 10mM dNTP Mix | 0.1 |
| 1M TMAC | 0.15 |
| 5X CES Buffer | 1 |
| Primers (**20, 10, 5, 2.5, 1μM** ) | **2** |
| Test DNA (10ng/μl) | 1 |
| 5U/μl GoTaq Hot Start Polymerase | 0.04 |
| **Total Volume** | **10μl** |

1. Perform gradient PCR amplification using the following PCR cycling conditions with a heated lid:
   1. 94°C for 7 minutes
   2. 94°C for 20 seconds
   3. **55-57°C** for 30 seconds
   4. 72°C for 2 minutes
   5. Repeat steps b-d for a total of 35 cycles
   6. 72°C for 5 minutes
   7. Hold at 4°C
2. Loading all 10μl, visualise the PCR products on a 2% agarose gel (with TAE buffer).
3. The optimal temperature (e.g. 56°C) and primer concentration (e.g. 10μM) yields the most product with least primer dimers. **These conditions will be used for all subsequent Multiplex PCRs in this protocol.**

**2.3.4 Multiplex PCR optimisation: DNA Input**

This optimisation step, varying the levels of DNA input (e.g. 10ng, 5ng, 2.5ng, 1.25ng, 0.625ng and 0.3125ng), allows you to assess the **minimal amount** of DNA that your set of multiplex primers require for optimal amplification (at the optimal conditions as determined in the previous step). This step is useful for working with patient clinical samples which may have low DNA yields. *This step may be optional if your biological DNA samples have high yields of DNA.*

1. For each panel of pooled primers, prepare the following reaction mix at differing DNA inputs, using the optimal primer concentration as determined above, on ice in a 200 μl thin wall PCR tube, or 96-well plate:

| **Reagent** | **Volume (μl)** |
| --- | --- |
| Ultra Pure Water | 1.71 |
| 5X Green GoTaq Flexi Buffer | 2 |
| 25mM MgCl_2_ | 2 |
| 10mM dNTP Mix | 0.1 |
| 1M TMAC | 0.15 |
| 5X CES Buffer | 1 |
| Primers (at optimal concentration as determined above) | 2 |
| Test DNA (**10ng, 5ng, 2.5ng, 1.25ng, 0.625ng, 0.3125ng** ) | 1 |
| 5U/μl GoTaq Hot Start Polymerase | 0.04 |
| **Total Volume** | **10μl** |

1. Perform PCR amplification using the following PCR cycling conditions with a heated lid, at the optimal annealing temperature as determined above:
   1. 94°C for 7 minutes
   2. 94°C for 20 seconds
   3. **56°C** for 30 seconds
   4. 72°C for 2 minutes
   5. Repeat steps b-d for a total of 35 cycles
   6. 72°C for 5 minutes
   7. Hold at 4°C
2. Loading all 10μl, visualise the PCR products on a 2% agarose gel (with TAE buffer). If only primer dimer band visible, multiplex PCR at the corresponding DNA input is not feasible.

**2.4 Multiplex bisulphite PCR**

Following optimisation of the multiplex primers, Multiplex Bisulphite PCRs on your biological samples’ DNA and methylated-control DNA can now be performed.

**2.4.1 Phosphorylation of multiplex primers**

The multiplex pooled primers are phosphorylated before proceeding to multiplex bisulphite PCR to assist in the ligation of Illumina indexing adaptors. Start with the pooled primers at twice the optimal concentration as determined above (2.3.3), such that if the optimal concentration was 10μM, start with 20μM pooled primers.

1. For each panel of pooled primers, prepare the following phosphorylation reaction mix, on ice in a 0.5ml tube:

| **Reagent** | **Volume (μl)** |
| --- | --- |
| Ultra Pure Water | 0.25 |
| 10x DNA ligase buffer | 5 |
| T4 Polynucleotide Kinase | 2 |
| 10mM ATP | 5 |
| 1M DTT | 0.25 |
| Pooled primers (at **2x the optimal concentration**) | 37.5 |
| **Total** | **50** |

1. Incubate at 37°C for 1 hour.
2. Top up the reaction mix with the following:

| **Reagent** | **Volume (μl)** |
| --- | --- |
| Ultra Pure Water | 18.75 |
| 10x DNA ligase buffer | 2.5 |
| T4 Polynucleotide Kinase | 1 |
| 10mM ATP | 2.5 |
| 1M DTT | 0.25 |
| **Total** | **25** |

1. Incubate at 37°C for 1 hour.
2. Your multiplex primers are now phosphorylated at the optimal concentration (e.g. 10uM) as determined by the optimisation PCRs.

***SAFE STOPPING POINT***

*If you do not plan to proceed to the Multiplex Bisulphite PCRs immediately, the protocol can be safely stopped here. Store the phosphorylated primers at -20°C.*

**2.4.2 Multiplex bisulphite PCRs with optimised conditions**

For Multiplex bisulphite PCRs on biological DNA samples, the PCR is run at 28 cycles only to reduce bias introduced in PCR process. The optimal DNA final concentration is 0.5ng/μl – 1ng/μl, subject to availability of DNA material.

1. For each bisulphite DNA sample, prepare the following reaction mix **in triplicate**, per panel of pooled primers, using the optimal conditions as determined above, on ice in a 200 μl thin wall PCR tube, or 96-well plate:

| **Reagent** | **Volume (μl)** |
| --- | --- |
| Ultra Pure Water | 2.065 |
| 5X **Colorless** GoTaq Flexi Buffer | 3 |
| 25mM MgCl_2_ | 3 |
| 10mM dNTP Mix | 0.15 |
| 1M TMAC | 0.225 |
| 5X CES Buffer | 1.5 |
| Phosphorylated Primers (at optimal concentration as determined above) | 3 |
| Bisulphite converted DNA | 2 |
| 5U/μl GoTaq Hot Start Polymerase | 0.06 |
| **Total Volume** | **15μl** |

1. Perform PCR amplification using the following PCR cycling conditions with a heated lid, at the optimal annealing temperature as determined above:
   1. 94°C for 7 minutes
   2. 94°C for 20 seconds
   3. **56°C** for 30 seconds
   4. 72°C for 2 minutes
   5. Repeat steps b-d for a total of **28 cycles**
   6. 72°C for 5 minutes
   7. Hold at 4°C

**2.4.3 PCR clean up**

A clean up follows the PCR step immediately, to remove primer dimer artefacts.

1. Remove PCR tubes/plate from the thermal cycler and combine the triplicate PCRs for each sample together into one 0.5ml tube or well (total volume = 45 μl per sample)
2. Pool PCR products from different multiplex panels together for **each sample** into one 0.5ml tube or in a 96 well plate. (E.g. total volume for two panels = 90 μl per sample)
3. Make up fresh 80% EtOH
4. Remove the AMPure XP beads from 4°C and let stand at room temperature for at least 30 minutes. Vortex the AMPure XP Beads until they are well dispersed.
5. To each tube/well of pooled PCR products, add Agencourt AMPure XP beads at a 1:1.6 (up to 1:2) ratio. For example, if the total volume of the pooled PCR products is 45μl (triplicate PCR, one panel), then add 72μl of beads.
6. Mix thoroughly by pipetting up and down. Incubate for 10 minutes at room temperature
7. Use a magnetic plate to aggregate the beads, wait 5 minutes until solution is clear, then remove and discard supernatant.
8. Wash with 200μl of 80% EtOH for 30 seconds. When adding EtOH, give the tubes/plate a little tap to disrupt the beads so that all the salt gets washed away. The beads aggregate back again quickly in EtOH. Remove and discard supernatant.
9. Repeat Wash (step 8)
10. Briefly centrifuge the tubes/plate so that excess EtOH goes to the bottom of the tube.
11. Air dry samples at room temperature for 5 minutes.
12. Add **9μl** Ultra Pure Water to each sample, vortex to resuspend beads.
13. Incubate at room temperature for 2 minutes.
14. Use a magnetic plate to aggregate the beads for 5 minutes, then transfer **7μl** of DNA into a new tube/plate, ready for ligation.

***SAFE STOPPING POINT***

*If you do not plan to proceed to the Library Preparation steps immediately, the protocol can be safely stopped here. Store the multiplex PCR products at -20°C.*

**2.5 Library Preparation**

**2.5.1 Sequencing adaptor ligation**

In this step, TruSeq Dual Index Adaptors are ligated to each sample.

1. Dilute the Illumina adaptor stock (typically 15μM) to 0.75μM (1:20 dilution).
2. Make up the following Ligation MasterMix using the recipe below (multiplied by number of samples):

| **Reagent** | **Volume (μl)** |
| --- | --- |
| UltraII Ligation MasterMix (NEB) | 3 |
| Ligation Enhancer (NEB) | 0.06 |
| Ultra Pure Water | 0.25 |
| **Total** | **3.31** |

1. To the beads cleaned 7μl PCR products, add 3.2μl of this Ligation Mastermix and 1μl of the Illumina adaptor (at 0.75μM each).
2. Incubate at 37°C for 30 minutes, then put this on ice.

Immediately procede to the next step

**2.5.2 Adaptor Ligation PCR**

A further round of 8-12 cycles of PCR was performed to amplify these libraries and incorporate the Illumina sequencing primers (P5 & P7).

1. For each library, prepare the following reaction mix on ice in a 200 μl thin wall PCR tube, or 96-well plate:

| **Reagent** | **Volume (μl)** |
| --- | --- |
| Ultra Pure Water | 12.8 |
| 5X Colorless GoTaq Flexi Buffer | 8 |
| 25mM MgCl_2_ | 9.6 |
| 10mM dNTP Mix | 0.4 |
| Primer P5 & P7 (Illumina) | 4 |
| Ligated DNA | 5 |
| 5U/μl GoTaq Hot Start Polymerase | 0.2 |
| **Total Volume** | **40** |

*Note: Store the rest of the ligated DNA at -20°C in case this step needs to be repeated.*

1. Perform adaptor ligation PCR amplification using the following PCR cycling conditions with a heated lid:
   1. 94°C for 5 minutes
   2. 97°C for 15 seconds
   3. 55°C for 30 seconds
   4. 72°C for 2 minutes
   5. Repeat steps b-d for a total of 8-12 cycles
   6. 72°C for 5 minutes
   7. Hold at 4°C
2. *Optional: Following PCR amplification, load and run 5μl of PCR products on 2% agarose gel to ensure multiple bands are seen: top band = library, intermediate bands = adaptor and lower band = primer dimer.*

**2.5.3 Library Purification**

Perform library purification immediately following adaptor ligation PCR**.**

1. Make up fresh 80% EtOH
2. Remove the AMPure XP beads from 4°C and let stand at room temperature for at least 30 minutes. Vortex the AMPure XP Beads until they are well dispersed.
3. Add 40μl (1:1 ratio) of Agencourt AMPure XP beads to each 40μl reaction mix
4. Mix thoroughly by pipetting up and down. Incubate for 10 minutes at room temperature
5. Use a magnetic plate to aggregate the beads, wait 5 minutes until solution is clear, then remove and discard supernatant.
6. Wash with 200μl of 80% EtOH for 30 seconds. When adding EtOH, give the tubes/plate a little tap to disrupt the beads so that all the salt gets washed away. The beads aggregate back again quickly in EtOH. Remove and discard supernatant.
7. Repeat Wash (step 8)
8. Briefly centrifuge the tubes/plate so that excess EtOH goes to the bottom of the tube.
9. Air dry samples at room temperature for 5 minutes.
10. Add **20μl** TruSeq Resuspension Buffer to each sample, vortex to resuspend beads.
11. Incubate at room temperature for 2 minutes.
12. Use a magnetic plate to aggregate the beads for 5 minutes, then transfer **18μl** of DNA into a new tube/plate.

***SAFE STOPPING POINT***

*If you do not plan to validate library immediately, the protocol can be safely stopped here. Store the cleaned adaptor ligated PCR products at -20°C.*

**2.6 Validate Library**

The following procedures outline our methodology for quality control analysis and quantification of multiplex bisulphite PCR libraries. At this stage, multiplex PCRs should be pooled together across different panels for **each individual sample**, with sequencing adaptors ligated per sample in a tube or in a plate format.

**2.6.1 Quantify libraries & Quality Control – Individual Libraries**

1. First quantify each individual library (e.g. per sample) using the Qubit dsDNA HS Assay Kit according to the manufacturer’s instructions. Choose a few individual libraries, ranging from the lowest and highest concentration libraries (based on Qubit results), and run them on the Agilent 4200 TapeStation system (D1000), to check library amount, size (~250bp based on amplicon size plus sequencing adaptors) and purity.

**2.6.2 Quantify libraries & Quality Control – Pooled Library**

1. Pool individual libraries at equal amounts into one 0.5ml tube. For one sequencing run, up to 96 libraries can be pooled together.

2. Run the pooled library (or libraries) on the Agilent 4200 TapeStation system (D1000). If primer dimer bands (<200bp) were observed, a second clean-up (1:1 Agencourt beads) should be performed and the quantification steps above repeated.

3. Quantify the pooled library using the Qubit dsDNA HS Assay Kit.

4. Dilute the pooled library to 10-20nM according to Qubit quantification

5. Quantify the pooled library using qPCR according to the KAPA Library Quantification Kit (Illumina)

6. Dilute the pooled library to 10nM according to KAPA qPCR results, ready for sequencing

***SAFE STOPPING POINT***

*If you do not plan to proceed to sequencing immediately, the protocol can be safely stopped here. Store the pooled library (or libraries) at -20°C.*

**2.7 Sequencing**

1. Prepare your pooled library for sequencing on Illumina MiSeq^TM^ or NextSeq^TM^ machines according to Illumina’s instructions.

2. Include sequencing controls (e.g. PhiX Control v3) in your sequencing run.

**2.8. Analysis of multiplex PCR sequencing data**

1. Use MethPanel (1) to preprocess and align reads to pre-defined regions (based on output of PrimerSuite) of the reference genome (hg19 build).

2. The associated MethPanel ShinyApp (https://github.com/thinhong/MethPanel) can be used to visualise the sequencing data (coverage, methylation, bias and epipolymorphism).

**References**

1. Luu PL, Ong P-T, Loc TTH, Lam D, Pidsley R, Stirzaker C, et al. MethPanel: a parallel pipeline and interactive analysis tool for multiplex bisulphite PCR sequencing to assess DNA methylation biomarker panels for disease detection. BioRxiv. 2020.

2. Johnston AD, Lu J, Ru KL, Korbie D, Trau M. PrimerROC: accurate condition-independent dimer prediction using ROC analysis. Sci Rep. 2019;9(1):209.

3. Lu J, Johnston A, Berichon P, Ru KL, Korbie D, Trau M. PrimerSuite: A High-Throughput Web-Based Primer Design Program for Multiplex Bisulfite PCR. Sci Rep. 2017;7:41328.

4. Ralser M, Querfurth R, Warnatz HJ, Lehrach H, Yaspo ML, Krobitsch S. An efficient and economic enhancer mix for PCR. Biochem Biophys Res Commun. 2006;347(3):747-51.
