# Supplementary material for: Comprehensive evaluation of targeted multiplex bisulphite PCR sequencing for validation of DNA methylation biomarker panels
Source: Clin Epigenetics. 2020 Jun 22;12:90. doi: 10.1186/s13148-020-00880-y (PMC7310104; doi:10.1186/s13148-020-00880-y)
Supplement: Supplementary file 3 — Additional file 3: Figure S2. Pre-sequencing optimisation of multiplex PCR primers - Prostate Cancer panels (PDF). (A) PCR products of singleplex amplification of 63 individual primer pairs from the prostate cancer panels run on 2% agarose gel. The gel shows the specificity of all the primer pairs and PCR products of the correct size (105-150 bp) with minimal primer dimer formation. (-) no template; (+) bisulphite-treated test DNA template (10ng), (L) 100 bp DNA Ladder. (B) Singleplex primers were pooled into their respective multiplex panels and the success of multiplex PCR reactions is shown at different primer concentrations (20 μM, 10 μM, 5 μM and 1 μM) and at an annealing temperature of 56 °C. (C) PCR products from the multiplex panel testing DNA input amounts from 10 ng, 5 ng, 2.5 ng, 1.25 ng of bisulphite-treated control DNA; (+) test DNA; (-) no template control. [file 13148_2020_880_MOESM3_ESM.pdf]

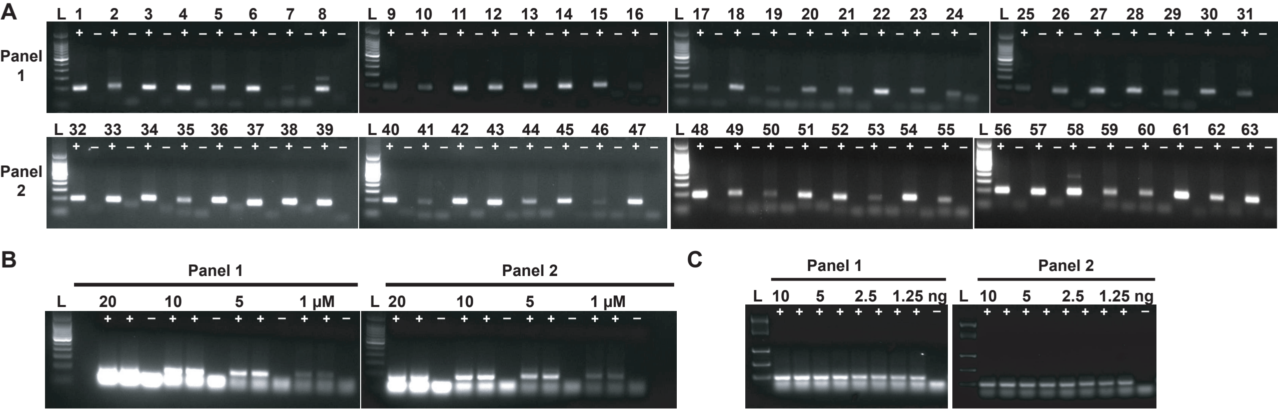

Figure S2. Pre-sequencing optimisation of multiplex PCR primers - prostate cancer panels. (A) PCR products of singleplex amplification of 63 individual primer pairs from the prostate cancer panels run on 2% agarose gel. The gel shows the specificity of all the primer pairs and PCR products of the correct size (105-150 bp) with minimal primer dimer formation. (-) no template; (+) bisulphite-treated test DNA template (10 ng), (L) 100 bp DNA Ladder. (B) Singleplex primers were pooled into their respective multiplex panels and the success of multiplex PCR reactions is shown at different primer concentrations (20 μM, 10 μM, 5 μM and 1 μM) and at an annealing temperature of 56 °C. (C) PCR products from the multiplex panel testing DNA input amounts from 10 ng, 5 ng, 2.5 ng, 1.25 ng of bisulphite-treated control DNA; (+) test DNA; (-) no template control.
