# Supplementary material for: Comprehensive evaluation of targeted multiplex bisulphite PCR sequencing for validation of DNA methylation biomarker panels
Source: Clin Epigenetics. 2020 Jun 22;12:90. doi: 10.1186/s13148-020-00880-y (PMC7310104; doi:10.1186/s13148-020-00880-y)
Supplement: Supplementary file 4 — Additional file 4: Figure S3. Technical Sensitivity and Coverage (PDF). Sensitivity of the multiplex PCR bisulphite sequencing assay was assessed using methylated-control DNA at 0%, 1% and 5% expected levels of methylation, across three separate sequencing runs. Three representative amplicons are shown. Observed methylation (%) (y-axis) is plotted against expected methylation (%) (x-axis). Down-sampling sequencing coverage at 100x, 1,000x, 10,000x and 100,000x shows that the sensitivity of detection of methylation improves with increased coverage. [file 13148_2020_880_MOESM4_ESM.pdf]

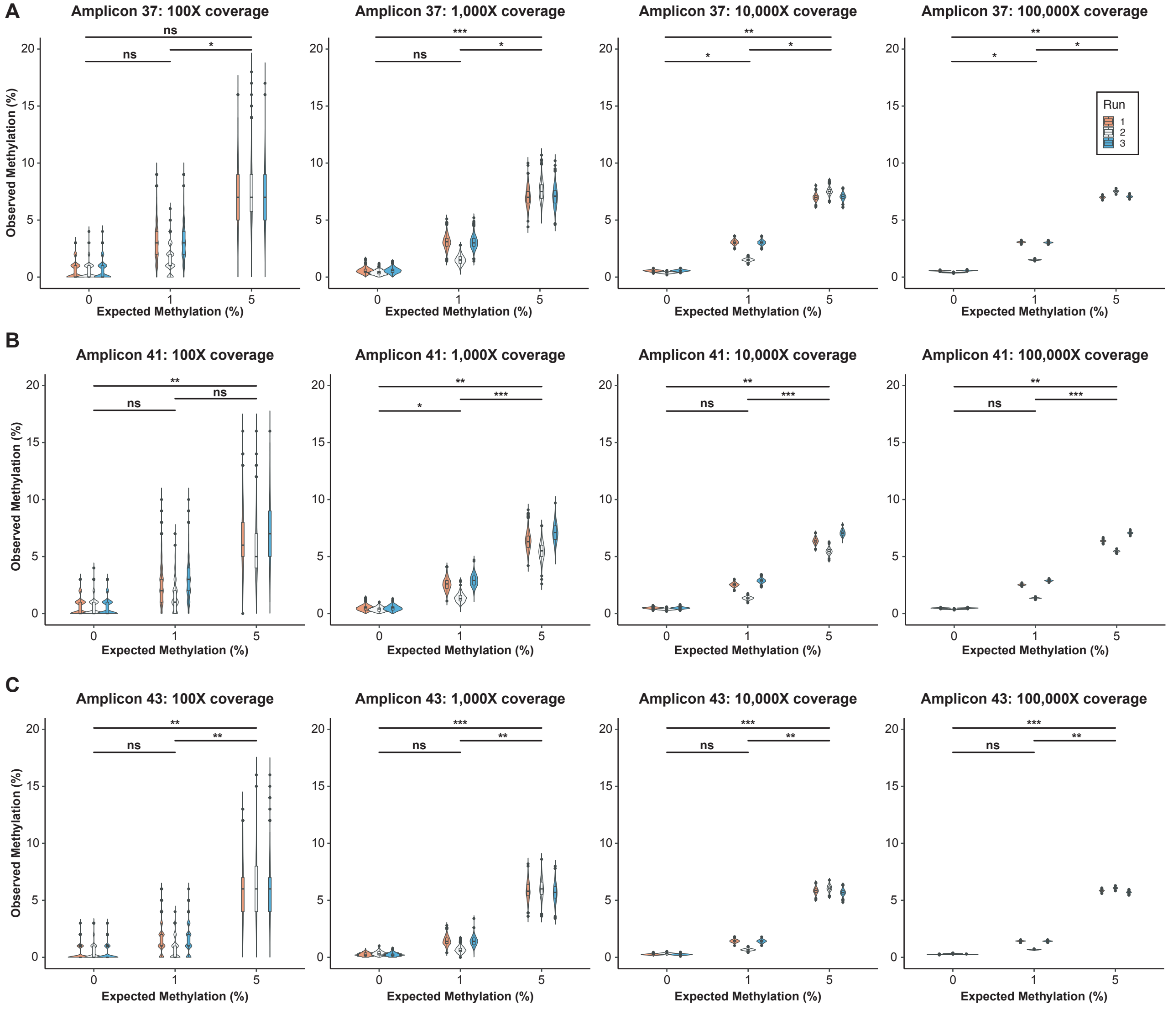

Figure S3. Technical Sensitivity and Coverage. Sensitivity of the multiplex PCR bisulphite sequencing assay was assessed using methylated-control DNA at 0%, 1% and 5% expected levels of methylation, across three separate sequencing runs. Three representative amplicons are shown. Observed methylation (%) (y-axis) is plotted against expected methylation (%) (x-axis). Down-sampling sequencing coverage at 100x, 1,000x, 10,000x and 100,000x shows that the sensitivity of detection of methylation improves with increased coverage.
