# Supplementary material for: Comprehensive evaluation of targeted multiplex bisulphite PCR sequencing for validation of DNA methylation biomarker panels
Source: Clin Epigenetics. 2020 Jun 22;12:90. doi: 10.1186/s13148-020-00880-y (PMC7310104; doi:10.1186/s13148-020-00880-y)
Supplement: Supplementary file 5 — Additional file 5: Figure S4. Platform comparison of MBPS and WGBS methylation data (PDF). (A) Correlation plots of absolute methylation levels of each CpG in the prostate cancer panels, as measured by multiplex bisulphite PCR (y-axis) and WGBS (x-axis), across each of the four cells (LNCaP, PrEC, CAF, NPF). (B) Correlation plots of relative methylation differences (LNCaP minus PrEC; CAF minus NPF cells) as measured by MBPS (y-axis) and WGBS (x-axis). Pearson’s correlation test was used to derive the correlation coefficient (r) and p-value (p). [file 13148_2020_880_MOESM5_ESM.pdf]

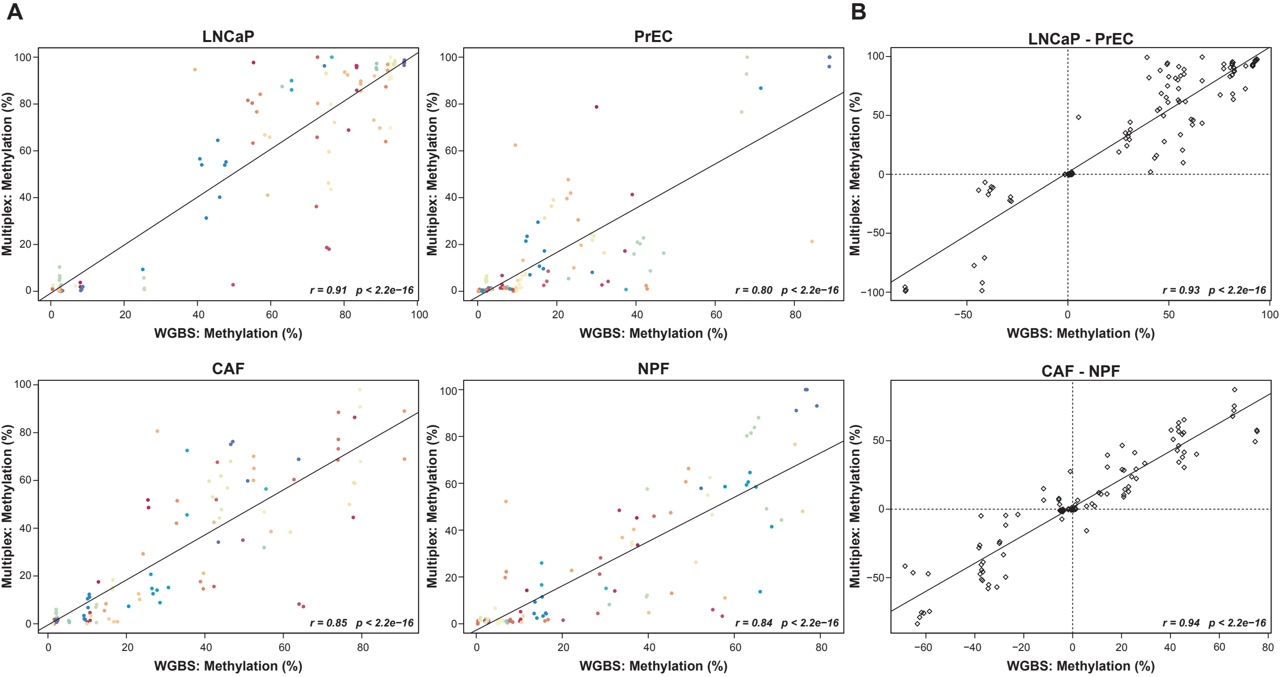

Figure S4. Platform comparison of the MBPS and WGBS methylation data. (A) Correlation plots of absolute methylation levels of each CpG in the prostate cancer panels, as measured by multiplex bisulphite PCR (y-axis) and WGBS (x-axis), across each of the four cells (LNCaP, PrEC, CAF, NPF). (B) Correlation plots of relative methylation differences (LNCaP minus PrEC; CAF minus NPF cells) as measured by MBPS (y-axis) and WGBS (x-axis). Pearson's correlation test was used to derive the correlation coefficient (r) and p-value (p).
