# Supplementary material for: Comprehensive evaluation of targeted multiplex bisulphite PCR sequencing for validation of DNA methylation biomarker panels
Source: Clin Epigenetics. 2020 Jun 22;12:90. doi: 10.1186/s13148-020-00880-y (PMC7310104; doi:10.1186/s13148-020-00880-y)
Supplement: Supplementary file 6 — Additional file 6: Figure S5. MBPS of bisulphite-treated FFPET clinical DNA (PDF). Tape Station electropherogram showing representative sequencing libraries from breast cancer (A) FFPET-derived DNA samples and (B) circulating cell-free DNA samples. The grey peak at ~250 bp represents the library (amplicon + sequencing adaptors), with peaks observed < 200 bp representing residual primer dimers. [file 13148_2020_880_MOESM6_ESM.pdf]

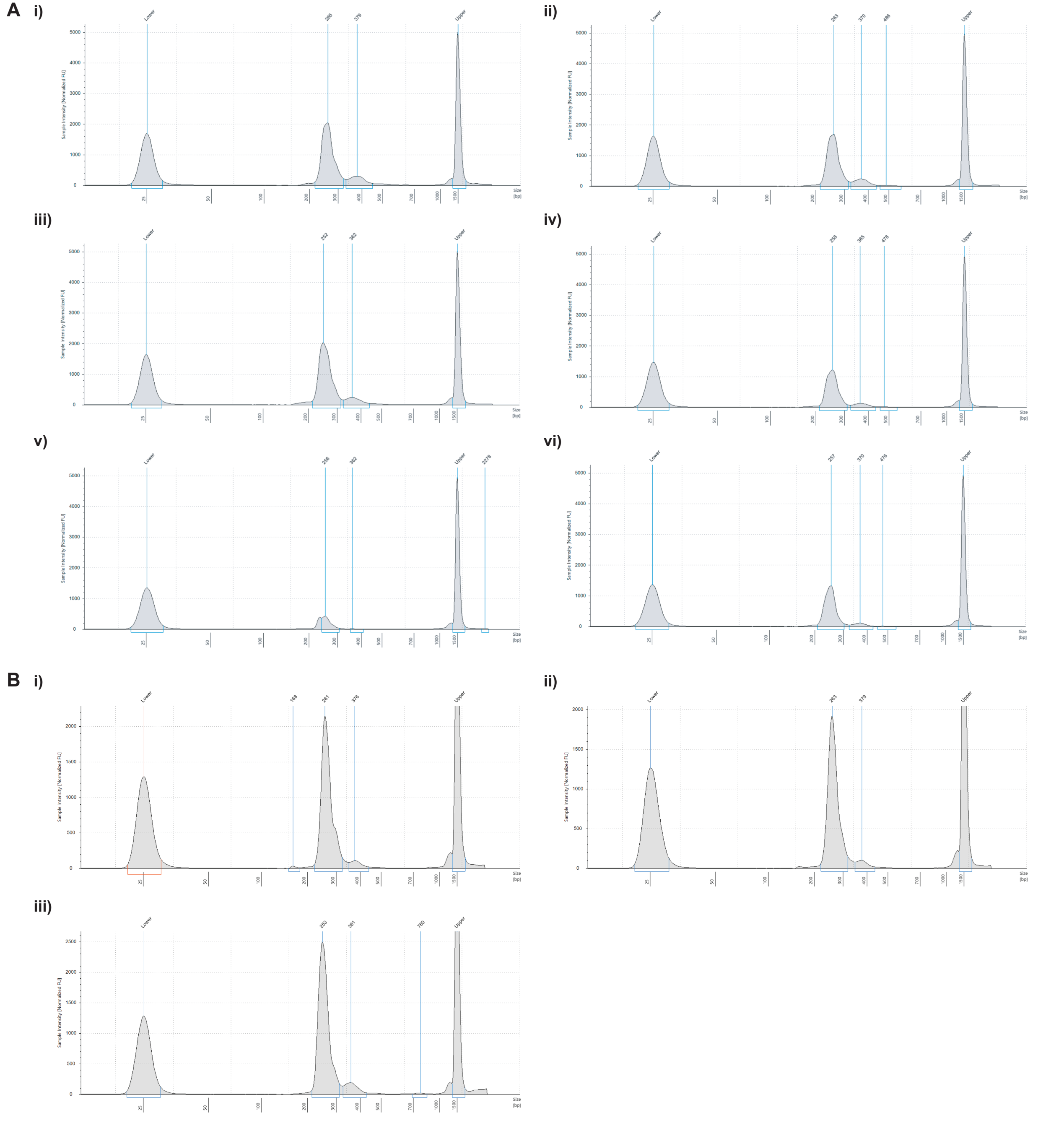

Figure S5. MBPS of bisulphite-treated clinical DNA. Tape Station electropherogram showing representative sequencing libraries from breast cancer (A) FFPET-derived DNA samples and (B) circulating cell-free DNA samples. The grey peak at ~250 bp represents the library (amplicon + sequencing adaptors), with peaks observed < 200 bp representing residual primer dimers.
